# OsMYB306‐OsRAV11 Regulates Resistance of Rice to Striped Stem Borer by Modulating Serotonin Biosynthesis

**DOI:** 10.1111/pbi.70680

**Published:** 2026-05-07

**Authors:** Jia‐Run Zhang, Long Wang, Yang‐Fan Chen, Xiao‐Hao Guo, Meng Jiang, Yuan‐Yuan Tan, Qing Wang, Qiu Qian, Angharad M. R. Gatehouse, Qing‐Yao Shu

**Affiliations:** ^1^ State Key Laboratory of Rice Biology & Breeding, and Zhejiang Provincial Key Laboratory of Crop Germplasm Innovation and Exploitation, The Advanced Seed Institute Zhejiang University Hangzhou China; ^2^ Hainan Institute Zhejiang University Sanya China; ^3^ Zhejiang University – Wuxi Xishan Joint Modern Agricultural Research Centre Zhejiang University Hangzhou China; ^4^ School of Natural and Environmental Sciences Newcastle University Newcastle upon Tyne UK

**Keywords:** OsMYB306, OsRAV11, rice, serotonin, striped stem borer, transcriptional regulation, tryptamine 5‐hydroxylase

## Abstract

Striped stem borer (SSB; *Chilo suppressalis* Walker) is one of the most destructive pests in rice production. Previous studies have demonstrated that SSB infestation induces transcription of *OsT5H* (tryptamine‐5‐hydroxylase) and biosynthesis of serotonin, a newly recognised phytohormone, and that disruption of serotonin biosynthesis significantly increases SSB resistance. However, the regulatory module modulating serotonin biosynthesis remains to be identified and characterised. Here, we reveal an OsMYB306‐OsRAV11 module that regulates *OsT5H* transcription and serotonin biosynthesis in response to SSB infestation in rice. OsMYB306 and OsRAV11 can bind to the *OsT5H* promoter and repress its transcription. In the module, OsRAV11 interacts with OsMYB306 and enhances its inhibitory effect on *OsT5H* transcription. CRISPR/Cas9‐generated knockout mutants (*myb306*, *rav11* and *myb306 rav11*) exhibited elevated *OsT5H* expression, increased serotonin accumulation and reduced SSB resistance. Conversely, *OsRAV11* overexpression reduced *OsT5H* transcription. Our findings establish a transcriptional regulatory framework for the biosynthesis of serotonin in response to SSB infestation. These findings inform the development of new strategies for producing SSB‐resistant rice by genome editing, potentially reducing reliance on chemical pesticides for SSB control.

## 1 Introduction

Rice ( *Oryza sativa* L.) is a globally significant staple crop and is the primary energy source for nearly half of the world's population (Thapa and Bhusal 2020). However, rice production is severely threatened by various pathogens and pest insects with global yield losses of up to 30% (Savary et al. 2019). Whilst rice plants have co‐evolved genes and pathways for resistance to some insect pests such as brown plant hopper (BPH) (Du et al. 2009), no germplasm showing sufficient levels of resistance to striped stem borer (SSB, *Chilo suppressalis* Walker), one of the most destructive pests in rice production (Fahad et al. 2015), has been identified to date. The exception to this is transgenic lines expressing Bt genes (Shu et al. 2000) and gene‐edited plants with mutants of the *OsT5H* gene (Lu et al. 2018). The *OsT5H* knockout mutants, on the other hand, although resistant to both BPH and SSB, are not suitable as new commercial varieties due to the presence of yield‐affecting lesion mimics in the leaves and sheaths when grown in the field (Lu et al. 2018; Wang et al. 2023). Therefore, SSB control has so far been heavily reliant on the extensive use of chemical pesticides, driving an urgent need to develop resistant varieties for increased rice production.

The *OsT5H* gene encodes tryptamine‐5‐hydroxylase, which converts tryptamine to serotonin (5‐hydroxytryptamine, 5‐HT) (Fujiwara et al. 2010). 5‐HT, a pineal hormone in animals, was first reported in plants in 1954 (Erland et al. 2015); it has since been shown to play important roles in plant growth and development, as well as in plant response to various environmental stimuli (Sun et al. 2025). In rice, SSB infestation can significantly increase serotonin accumulation (Ishihara et al. 2008). Our previous studies demonstrated that infestation by both BPH and SSB led to increased *OsT5H* transcription, and hence increased serotonin biosynthesis and accumulation in rice (Lu et al. 2018). In that same study we revealed that the induced *OsT5H* transcription was blocked in BPH‐resistant rice varieties, and that the *OsT5H* knockout exhibited significantly enhanced rice resistance to both BPH and SSB. We further demonstrated that the *OsT5H* knockout does not disrupt the innate defence response of rice plants to SSB infestation, indicating it is a deficiency of serotonin that enhanced SSB resistance rather than the effect of the *OsT5H* knockout itself on the plant defence response (Wang et al. 2023). Findings from these studies suggest that rice varieties with enhanced resistance could be developed by disrupting the signalling pathway of SSB/BPH‐induced serotonin biosynthesis, which would keep the basal serotonin biosynthesis sufficient for normal plant growth.

Although numerous studies have shown that serotonin biosynthesis and accumulation can be induced by various biotic and abiotic stresses, its underpinning molecular mechanisms remain mostly elusive in plants (Sun et al. 2025). In cassava, Wei et al. (2018) demonstrated that two cassava apetala2/ethylene response factor (AP2/ERF) gene family members, MeRAV1 and MeRAV2, directly bind to the promoter of *T5H* and activate its transcription (Wei et al. 2018). They further showed that MeRAV1 and MeRAV2 were targeted by the coat protein of cassava common mosaic virus to subvert the immunity of cassava against the virus (Wei et al. 2024). In rice, Sun et al. (2022) demonstrated that UV‐B stress induces *OsbZIP18* transcription, and that OsbZIP18 activates serotonin biosynthesis genes, including *tryptophan decarboxylase 1* (*OsTDC1*), *tryptophan decarboxylase 3* (*OsTDC3*) and *OsT5H* by directly binding to the ACE‐containing or G‐box *cis*‐elements in their promoters (Sun et al. 2022). These studies indicate that a set of specific transcriptional factors and partner proteins are responsible for the induction of serotonin biosynthesis by a given stress, such as SSB infestation.

With the aim of developing a strategy for breeding SSB resistant rice varieties by modulating *OsT5H* transcription and serotonin biosynthesis, this study focused both on the identification of transcription factors that regulate *OsT5H* transcription in response to SSB infestation and elucidating the underlying mechanisms involved. We first identified an R2R3‐MYB transcription factor, OsMYB306, that could directly bind to the *OsT5H* promoter, and then identified an OsMYB306‐interacting transcription factor, OsRAV11, that could also bind to the *OsT5H* promoter. We further revealed an OsMYB306‐OsRAV11 regulatory module that synergically represses *OsT5H* transcription to modulate serotonin biosynthesis and consequently resistance to SSB in rice.

## 2 Results

### 2.1 OsMYB306 Binds to the CAACCA Motif in the *OsT5H* Promoter

To identify transcription factors that regulate *OsT5H* transcription, a yeast one‐hybrid (Y1H) library screening was performed using the promoter of *OsT5H* as bait and a rice cDNA library as prey. More than 100 positive clones were detected on plates with a synthetic dropout (SD) medium without leucine but supplemented with 200 ng/mL Aureobasidin A (AbA). Subsequent sequencing of the positive clones revealed cDNA sequences that were part of a total of 32 genes, nine of which were classified as transcription factors (Table S1). Further Y1H analyses for all these 9 transcription factors demonstrated that only *LOC_08g33940* could actually bind to the *OsT5H* promoter (Figure S1).

To maximise the possibility of identifying potential transcription factors that bind to the *OsT5H* promoter, we performed an *in silico* analysis of the *OsT5H* promoter region using PlantRegMap (http://plantregmap.gao‐lab.org/). The results revealed 48 transcription factors with putative binding affinity to the *OsT5H* promoter (Table S2). Joint analysis of the transcription factors revealed by PlantRegMap analysis (48) and by Y1H library screening (9) identified *LOC_Os08g33940* as the sole shared gene. Hence, we focused on the characterisation of *LOC_Os08g33940*.

*LOC_Os08g33940* has previously been reported to be a member of the MYB or MYB‐like gene family in a genome‐wide classification and expression analysis of MYB transcription factor families in rice (Katiyar et al. 2012). This gene is designated as myb‐related protein 306 in the NCBI (LOC9272734 myb‐related protein 306 [Oryza sativa Japonica Group (Japanese rice)] ‐ Gene ‐ NCBI). For simplicity, we have designated this gene as *OsMYB306*.

**FIGURE 1:**
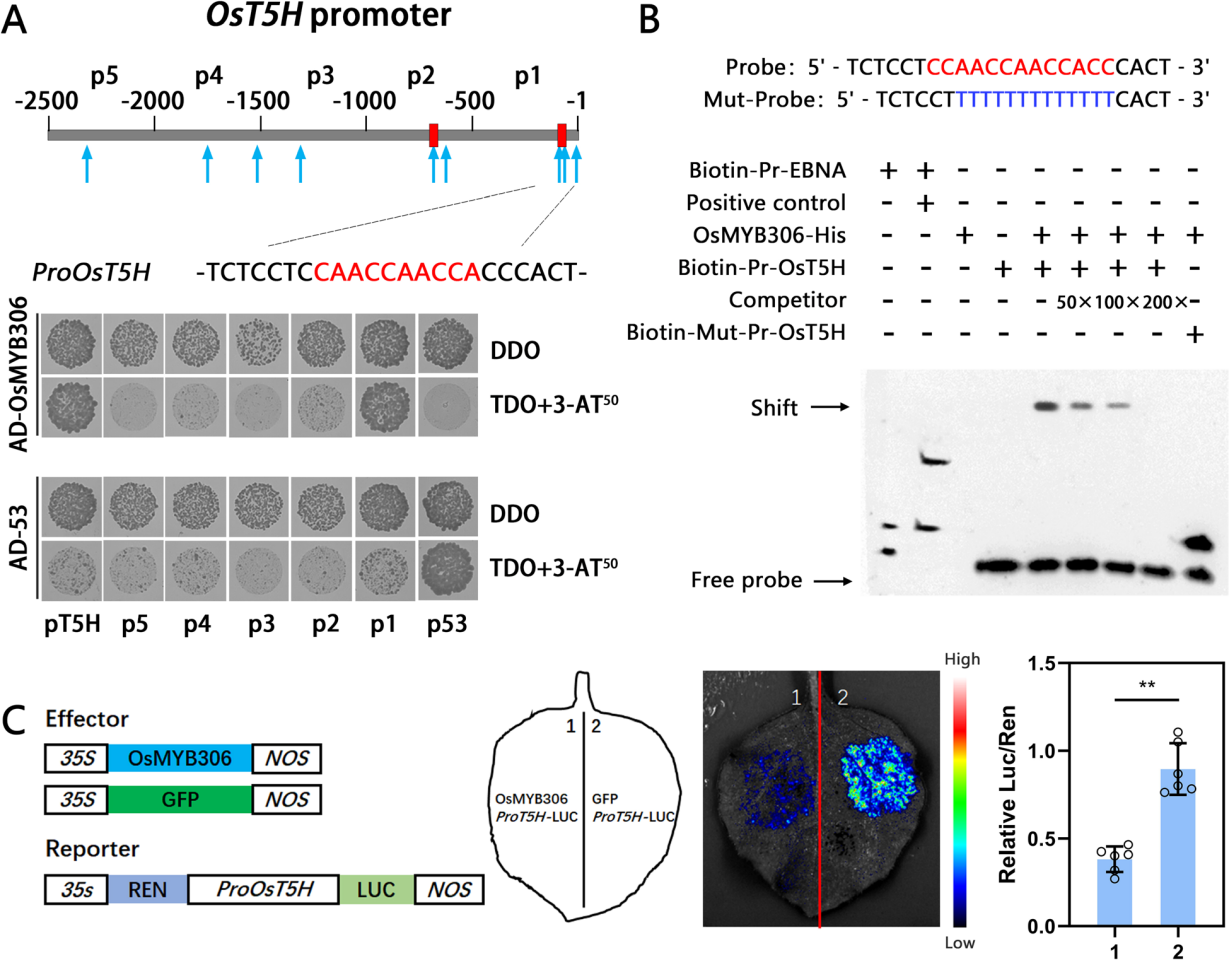
OsMYB306 binds to the *OsT5H* promoter. (A) *Upper*: Schematic diagram of the *OsT5H* promoter. Blue arrows indicate the location of potential MYB binding motifs revealed by PlantCARE analysis. Red squares represents two potential binding sequences predicated by PlantRegMap. *Bottom*: Yeast one‐hybrid assays showing OsMYB306 binding to the promoter fragments of *OsT5H*. The basal concentration of 3‐AT was 50 mM. AD‐53 and pHIS2‐p53 were used as a positive control. (B) Interaction of the OsMYB306‐His protein with the *OsT5H* promoter in electrophoretic mobility shift assays (EMSA). Positive control, EBNA extract; Biotin‐Pr‐EBNA, was used as a probe for positive control. Unlabeled probes were used as competitors. ‘+’ and ‘−’ indicate the presence or absence of proteins or probes in the mixtures. (C) Relative LUC activities in *N. benthamiana* leaf segments transformed with the *ProOsT5H‐LUC* together with the indicated effector constructs. Means and standard deviations were calculated from the results of six biological repetitions. Asterisks indicate significant differences of relative Luc/Ren ratio between the indicated effector constructs. (Student's *t*‐test, **p < 0.01).

To confirm that OsMYB306 binds to the *OsT5H* promoter, a series of in vitro and in vivo experiments were performed. First, to identify the potential sequence segments to which OsMYB306 might bind, PlantCARE analysis was performed for the *OsT5H* promoter, revealing the presence of 9 CAACCA motifs (Figure 1A, blue arrows). Second, to identify the specific OsMYB306 binding sites, several vectors (pHIS2‐p1 ~ −p5) with a 500‐bp segment of the *OsT5H* promoter (p1 ~ p5, Figure 1A) were constructed. Y1H assays to test their interaction with OsMYB306 demonstrated that only the yeast strain co‐expressing pHIS2‐p1 and AD‐OsMYB306 was able to grow on a TDO medium supplemented with 50 mM 3‐amino‐1,2,4‐triazole (3‐AT), similar to the positive control (pHIS2‐p53 and AD‐53) (Figure 1A). In contrast, yeast strains co‐expressing pHIS2‐p2 ~ −5 and AD‐OsMYB306, as well as those co‐expressing pHIS2‐p1 and AD‐53 (negative control), failed to grow under these same conditions (Figure 1A). These results suggest that OsMYB306 specifically binds to motifs within the p1 segment, which indeed hosts two partially overlapped CAACCA motifs (−47 bp ~ −38 bp) (Figure 1A, red square).

The interaction between OsMYB306 and the *OsT5H* promoter was further investigated through electrophoretic mobility shift assays (EMSA) using a recombinant OsMYB306‐His fusion protein. A distinct DNA‐protein complex was observed when a CAACCA‐containing oligonucleotide was used as a labelled probe. However, complex formation was reduced with increasing amounts of competitor (unlabeled probe) (Figure 1B). Furthermore, no DNA‐protein complex was detected when a mutated probe was used (Figure 1B). These results confirmed that the binding of OsMYB306 to the *OsT5H* promoter requires the CAACCA sequence.

Further studies were carried out to test whether OsMYB306 regulates *OsT5H* transcription in vivo using dual‐LUC reporter assays in *N. benthamiana* leaves. The results demonstrated that luciferase activity in regions co‐transformed with *35S::OsMYB306* and *ProOsT5H‐LUC* was significantly lower than those co‐transformed with *35S::GFP* and *ProOsT5H‐LUC* (negative control) (Figure 1C). These results demonstrated that OsMYB306 can repress *OsT5H* transcription in vivo, supporting its role as a transcriptional repressor.

Collectively, the above results demonstrate that OsMYB306 binds to the CAACCA motif in the promoter of *OsT5H* and negatively regulates its transcription in vivo.

In the absence of information either on *LOC_Os08g33940* (*OsMYB306*) or the protein (OsMYB306) it encodes, a series of experiments was performed to better characterise them. Bioinformatic analysis showed that OsMYB306 belongs to the R2R3‐type MYB family, which is distinguished by the presence of two conserved MYB DNA‐binding domains. Subcellular localisation assays showed that OsMYB306 was localised to the nucleus (Figure S2) in rice protoplasts co‐transfected with *35S::OsMYB306‐GFP* or *35S::GFP* and *NLS‐mCherry* plasmids.

### 2.2 *OsMYB306* Knockout Increases *OsT5H* Transcription and Serotonin Accumulation

**FIGURE 2:**
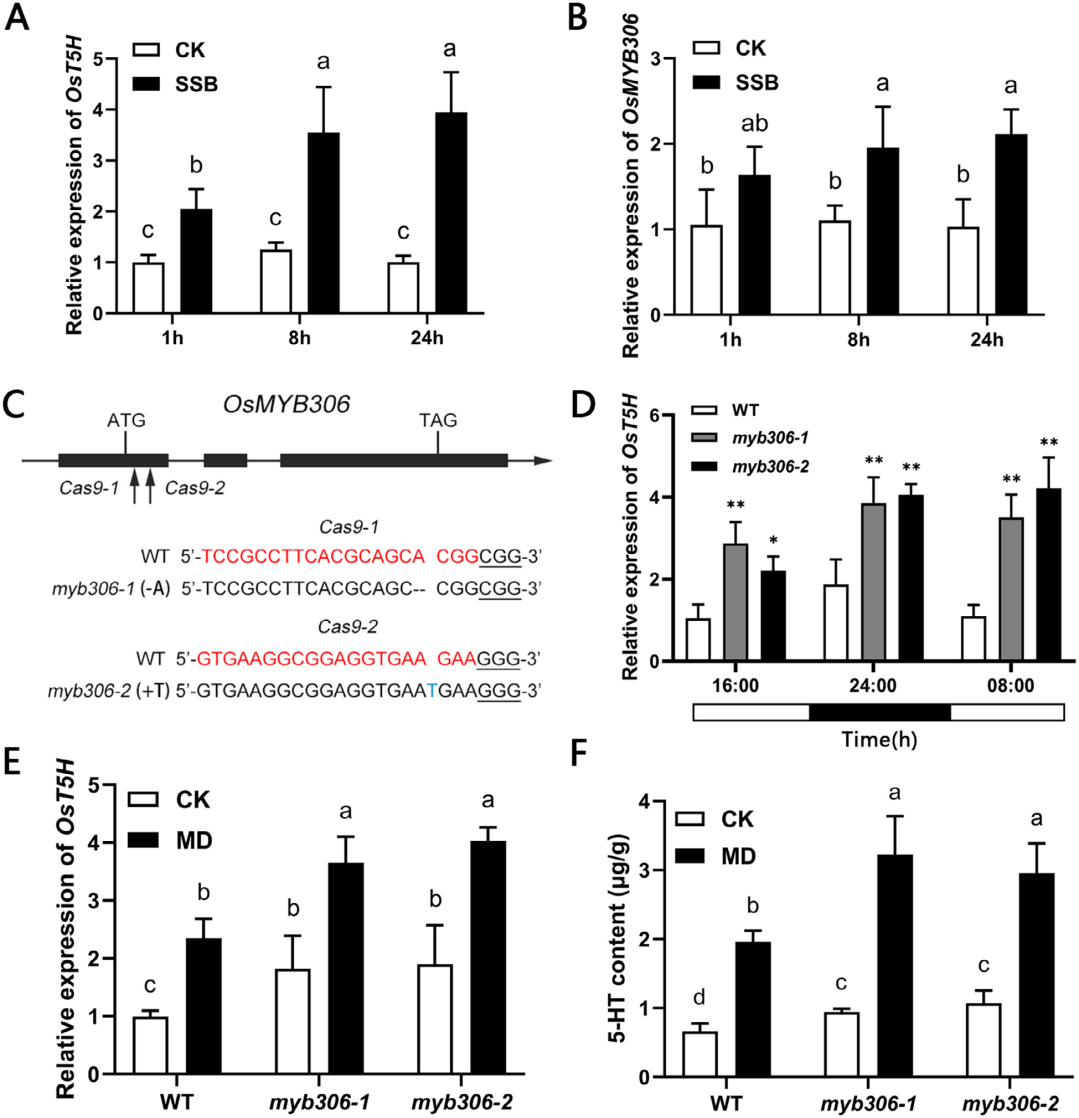
Knockout of *OsMYB306* increases *OsT5H* transcription and serotonin accumulation. (A and B) RT‐qPCR analysis of *OsT5H* (A) and *OsMYB306* (B) expression in 2‐month‐old wild‐type rice plants at 1 h, 8 h and 24 h after the onset of SSB feeding. The control group (CK) without feeding was sampled at the same time points. The expression levels of each gene, normalised to *Actin* expression, are presented relative to the CK, which is set to 1. Data are shown as mean ± SD of three biological replicates. Five plants in an individual block represent one replicate. Different letters indicate significant difference at *p* < 0.05 level (Student's *t*‐test). (C) CRISPR/Cas9‐mediated targeted mutagenesis of *OsMYB306*. *Upper*, a schematic diagram of *OsMYB306*, exons being represented by black boxes and introns by line; the CRISPR/Cas9 target sites are indicated by arrows. *Bottom*, nucleotide sequences flanking the PAM site (nucleotides underlined) in the two *OsMYB306* knockout lines *myb306‐1* and *myb306‐2*, and the wild‐type (WT) plants. (D) RT‐qPCR analysis of *OsT5H* in 2‐week‐old seedlings of wild‐type, *myb306‐1* and *myb306‐2*. Data are shown as mean ± SD of three biological replicates. Asterisk indicates significant difference between *myb306* and wild‐type (Student's *t*‐test, *p < 0.05, **p < 0.01). (E) RT‐qPCR analysis of *OsT5H* in 4‐week‐old wild‐type, *myb306‐1* and *myb306‐2*. Plants in the treatment group were subjected to mechanical damage (MD) using a hole puncher, followed by a 24‐h incubation period before being sampled alongside the control group (CK). Data are shown as mean ± SD of three biological replicates. Different letters indicate significant difference at *p* < 0.05 level (Student's *t*‐test). (F) 5‐HT levels in rice plants as treated in (C). The quantification was performed using high‐performance liquid chromatography (HPLC). Data are shown as mean ± SD of three biological replicates. Different letters indicate significant difference at *p* < 0.05 level (Student's *t*‐test).

To elucidate the biological function of OsMYB306 and its regulatory role in the defence response, we first examined the dynamic expression patterns of *OsMYB306* and its downstream target *OsT5H* in response to SSB infestation. In 2‐month‐old rice plants, *OsT5H* expression increased significantly at 1 h and reached approximately 4‐fold induction at 24 h post feeding onset (Figure 2A). *OsMYB306* expression was also induced by SSB feeding, with transcript levels increasing at 1 h and remaining around 2‐fold at 24 h (Figure 2B).

To further investigate the function of OsMYB306 on *OsT5H* transcription and also to elucidate its role in serotonin biosynthesis in rice plants, two *OsMYB306* knockout mutants were generated by CRISPR/Cas9‐mediated target mutagenesis (Figure 2C). Sequencing of the targeted genomic regions showed that *myb306‐1* and *myb306‐2* contained an adenine (A) deletion and a thymine (T) insertion, respectively. Both mutations resulted in frameshifts and truncated proteins (Figure S3). Given that *OsT5H* transcription is sensitive to the growing conditions of plants, particularly day and night (Wei et al. 2016), *OsT5H* mRNA abundance was measured at 3 time points across a 24‐h period in 2‐week‐old seedlings of the wild‐type (WT) parental cultivar Jiahua No. 1 and its two *OsMYB306* knockout mutants. The results showed that *OsT5H* mRNA was significantly more abundant in both *myb306‐1* and *myb306‐2* than in the WT at all time points (Figure 2D), supporting the LUC/REN observation that OsMYB306 acts as a transcriptional repressor of *OsT5H*.

When SSB larvae bite rice plants, it causes mechanical damage (MD) and delivers salivary effectors into plants. To examine the effects of MD on *OsT5H* transcription, we investigated *OsT5H* mRNA levels in WT rice plants either treated with MD, SSB mock feeding (MD + SSB saliva), or directly infested with SSB. The results showed that all three treatments significantly increased *OsT5H* transcription compared to the control plants. However, there were no differences between these treatments (Figure S4). This finding supports the use of MD as an alternative to SSB feeding in subsequent experiments related to transcriptional responses.

To determine the mutational effects of *OsMYB306*, we first examined *OsT5H* transcription in WT and mutant plants. MD treatment induced *OsT5H* expression in both mutants compared with WT, but caused a greater increase in *myb306‐1* and *myb306‐2* than that in the WT (Figure 2E).

To determine the effects of *OsT5H* mutation on serotonin biosynthesis, changes in serotonin content in the WT and mutant rice plants were measured using high‐performance liquid chromatography (HPLC). Consistent with the elevated *OsT5H* mRNA levels, the serotonin content was significantly higher in the two mutants than in the WT, particularly in those subjected to MD (Figure 2F). The serotonin content was 3.23 μg/g and 2.96 μg/g in *myb306‐1* and *myb306‐2*, respectively, significantly greater than that in the WT plants (1.96 μg/g) 24 h post MD.

The findings that the transcription of *OsT5H* and the serotonin content were significantly greater in *myb306‐1* and *myb306‐2* than in the WT, either in the presence or absence of MD, are consistent with the dual‐LUC reporter assays, which suggested that OsMYB306 functions as a transcriptional repressor of *OsT5H* (Figure 1C).

### 2.3 OsMYB306 Interacts With the Transcription Repressor OsRAV11

To elucidate the in‐depth mechanism underlying OsMYB306‐mediated regulation of *OsT5H* during SSB infestation in rice, we performed an immunoprecipitation–mass spectrometry (IP‐MS) analysis using protein extracts from rice tissues, including those collected 24 h after mechanical damage and SSB infestation. A total of 652 proteins were identified following the exclusion of results from the control group. Since our aim was to identify potential transcriptional regulators that might interact with OsMYB306, we focused on 16 proteins annotated as transcription factors (Table S3).

We hypothesised that potential OsMYB306‐interacting transcription factors would also respond to SSB damage, hence we analysed the RNA‐seq data of rice plants before and after SSB infestation (Wang et al. 2023). The results indicated that only *LOC_Os01g49830* was responsive (Figure S5), with transcript levels being significantly greater in SSB infested plants (277, 207, 125 FPKM, on average 1, 3, 8 h post infestation) than in non‐infested plants (20, 15, 12 FPKM, on average 1, 3, 8 h post infestation).

*LOC_Os01g49830* is annotated to encode a RAV (RELATED TO ABI3 AND VP1) family protein, OsRAV11. RAV proteins contain two distinct DNA‐binding domains: an APETALA2 (AP2) domain at the N‐terminus and a B3 domain at the C‐terminus. Previous studies have identified several RAV family transcription factors in *Arabidopsis*, including *AtRAV1* and *AtRAV2*, which exhibited transcriptional repressive activity (Ikeda and Ohme‐Takagi 2009). Further experiments revealed that this repressive function is mediated by a conserved short peptide motif, R/KLFGV (Ikeda and Ohme‐Takagi 2009). Through homology alignment, we identified OsRAV11 in rice as a homologue of AtRAV1 (AT1G13260) and confirmed the presence of a conserved repression domain RLFGV at the C‐terminal region (Figure S6). In rice, OsRAV11 has been demonstrated to play a role in reproductive development and its knock‐out resulted in elongated carpels (Osnato et al. 2020). In this study, *OsRAV11* was identified as a candidate protein interacting with OsMYB306 based on IP‐MS results. Subcellular localisation assays showed that OsRAV11‐GFP accumulated in the nucleus (Figure S7), consistent with its predicted role as a transcription factor.

**FIGURE 3:**
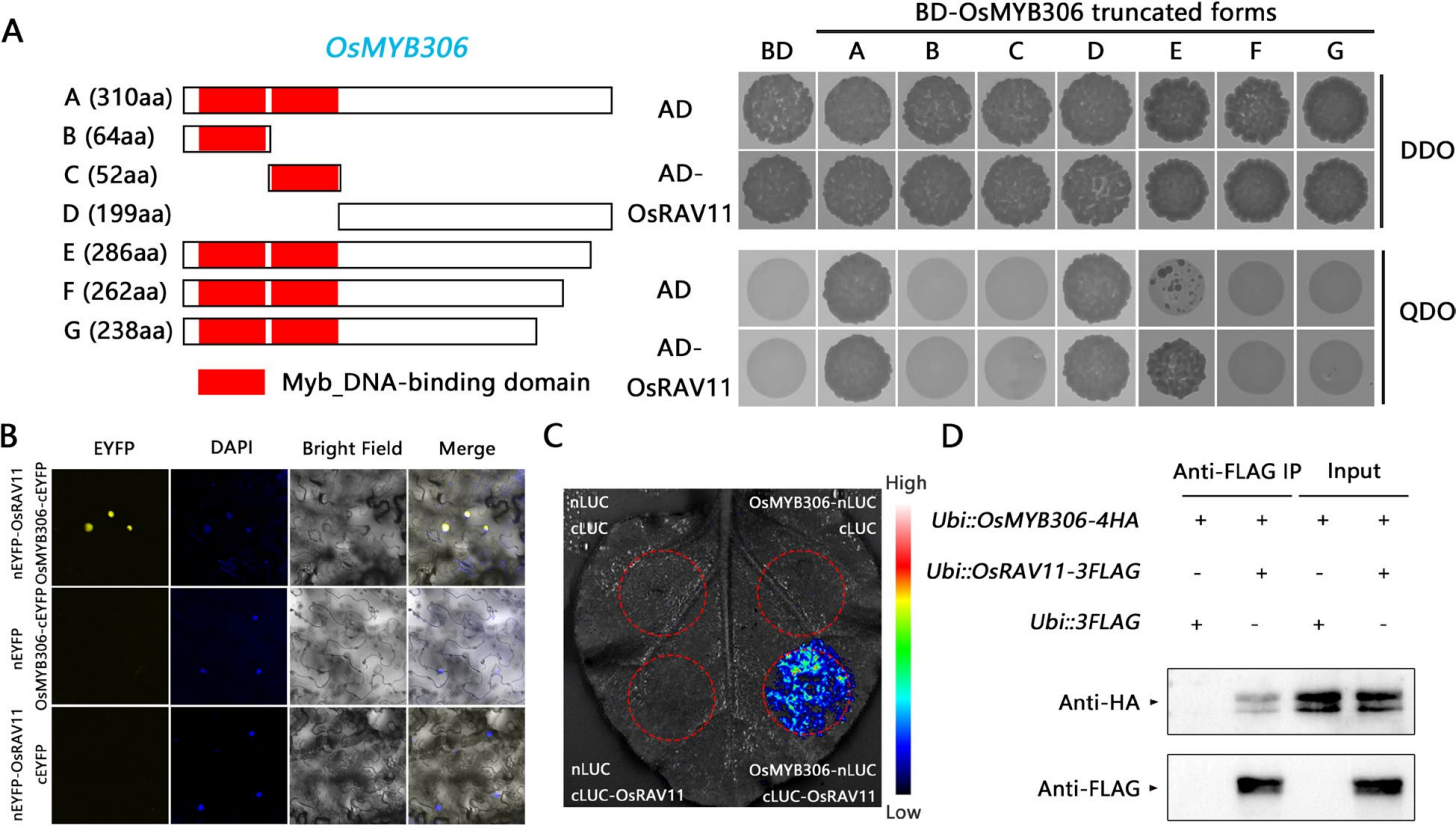
OsMYB306 interacts with OsRAV11. (A) Yeast two‐hybrid assay evaluating the interaction between OsRAV11 and various OsMYB306 truncated proteins. Schematic diagrams of OsMYB306 truncated proteins (A–G) that were fused to BD (empty vector). The full‐length OsMYB306 protein contains two Myb_DNA‐binding domains, which are denoted by red squares in the diagram. (B) BiFC assay of the interaction between OsMYB306 and OsRAV11 in *N. benthamiana* leaves. DAPI, fluorescence of 4′,6‐diamino‐2‐phenylindole; Merge, merged EYFP, DAPI and bright field images. (C) LCI assay of the interaction between OsMYB306 and OsRAV11. The indicated fusion pairs were co‐expressed in *N. benthamiana* leaves. (D) The interaction between OsMYB306‐4HA and OsRAV11‐3FLAG shown by Co‐IP in rice protoplasts. Total proteins were extracted and immunoprecipitated by anti‐FLAG magnetic beads. The input and immunoprecipitated proteins were detected by anti‐FLAG or anti‐HA antibody.

The interaction of OsRAV11 with OsMYB306 was confirmed in vitro by one‐to‐one Y2H assays. As strong self‐activation was observed when full‐length OsMYB306 was used, several BD constructs with truncated forms of OsMYB306 were generated (Figure 3A). OsMYB306 contains two MYB‐DNA binding domains; however, BD constructs with either one of the two domains did not show any self‐activation, and neither did they interact with OsRAV11 (Figure 3A, column B, C). In contrast, a fragment (286–310 aa) at the C‐terminal end appeared to be crucial for OsMYB306 self‐activation, whilst a different fragment near the C‐terminal region (262–286 aa) was essential for its interaction with OsRAV11(Figure 3A).

To further characterise the interaction between OsMYB306 and OsRAV11, a series of complementary studies were performed. Bimolecular fluorescence complementation (BiFC) assays in *N. benthamiana* leaves demonstrated their interaction in the nucleus in vivo (Figure 3B). Luciferase complementation imaging (LCI) analysis showed strong luciferase signals when they were co‐expressed (OsMYB306‐nLUC and cLUC‐OsRAV11) (Figure 3C). This interaction was further confirmed by swapping the luciferase fusions between the two proteins in tobacco leaves. Furthermore, we validated the interaction between OsMYB306 and OsRAV11 in rice protoplasts using co‐immunoprecipitation (Co‐IP). Expression vectors for *Ubi::OsMYB306‐4HA* and *Ubi::OsRAV11‐3FLAG*, as well as the control vector *Ubi:3FLAG*, were constructed and used for transformation into rice protoplasts. Tagged proteins were enriched using anti‐Flag magnetic beads for Western blot analysis. The results showed the co‐precipitation of OsMYB306 with OsRAV11 in protoplasts transfected with *Ubi::OsMYB306‐4HA* and *Ubi::OsRAV11‐3FLAG*, but not in those transfected with *Ubi::OsMYB306‐4HA* and *Ubi::3FLAG* (Figure 3D). Collectively, these data clearly demonstrate that OsMYB306 physically interacts with OsRAV11 in rice plants.

### 2.4 OsRAV11 Binds to the *OsT5H* Promoter and Cooperatively Represses Its Transcription With OsMYB306

**FIGURE 4:**
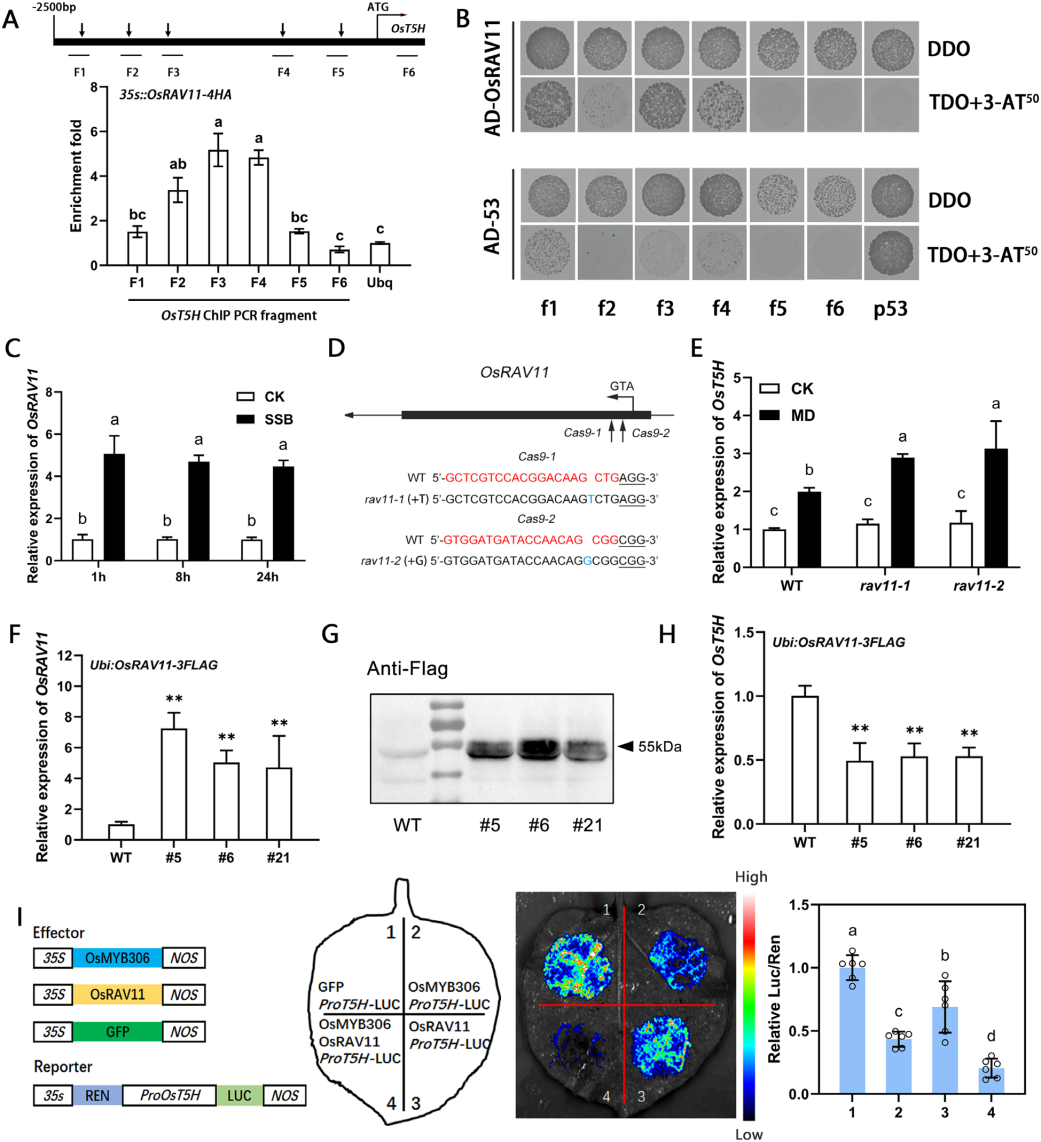
OsRAV11 Binds to the *OsT5H* promoter and cooperatively represses its transcription with OsMYB306. (A) ChIP‐qPCR analysis of OsRAV11 binding to the promoter of *OsT5H*. *Upper*, a schematic diagram of *OsT5H* and its promoter. The arrows indicate the location of CAACA motifs, and F1‐F6 are six fragments in ChIP‐PCR. *Bottom*, ChIP‐PCR results, data with different letters are significantly different at *p* < 0.05 level. Error bars indicate SD. (B) Yeast one‐hybrid assays showing OsRAV11 binding to the promoter fragments of *OsT5H*. The basal concentration of 3‐AT was 50 mM. AD‐53 and pHIS2‐p53 were used as a positive control. (C) RT‐qPCR analysis of *OsRAV11* in 2‐month‐old wild‐type plants at 1, 8 and 24 h after the onset of SSB feeding. The CK without feeding was sampled at the same time points. Data are shown as mean ± SD of three biological replicates. Five plants in an individual block represent one replicate. Different letters indicate significant difference at *p* < 0.05 level (Student's *t*‐test). (D) CRISPR/Cas9‐mediated targeted mutagenesis of *OsRAV11*. (E) RT‐qPCR analysis of *OsT5H* in 4‐week‐old wild‐type, *rav11‐1* and *rav11‐2* plants. Plants in the treatment group were subjected to mechanical damage using a hole puncher, followed by a 24‐h incubation period before being sampled alongside the control group. Data are shown as mean ± SD of three biological replicates. Different letters indicate significant difference at *p* < 0.05 level (Student's *t*‐test). (F and H) RT‐qPCR analysis of *OsRAV11* and *OsT5H* in independent 2‐week‐old T2 *Ubi:OsRAV11‐3FLAG* Lines. The levels of *OsRAV11* and *OsT5H* expression, normalised to *Actin* expression, are shown relative to the level in wild‐type plants, which was set to 1. Data are shown as mean ± SD of three biological replicates. Asterisks indicate significant differences between wild‐type and *Ubi:OsRAV11‐3FLAG* Plants (Student's *t*‐test, **p < 0.01). (G) Validation of the OsRAV11‐3Flag fusion protein using an Anti‐Flag antibody. (I) Relative LUC activities of the *ProOsT5H‐LUC* reporters co‐transformed with the indicated effector constructs. Means and standard deviations were calculated from the results of six biological replicates. Different letters indicate significant difference at *p* < 0.05 level (Student's *t*‐test). Error bars indicate SD.

Members of the RAV family possess highly conserved AP2 or B3 DNA binding domains (Chen et al. 2021). A previous study demonstrated that in *Arabidopsis*, the AP2 and B3‐like domains recognise the CAACA and CACCTG motifs, respectively (Kagaya et al. 1999). To explore the relationship between OsRAV11 and *OsT5H*, we analysed the cis‐regulatory elements in the *OsT5H* promoter and identified five CAACA motifs, but no CACCTG motifs. To identify which CAACA motif OsRAV11 binds to, ChIP‐qPCR assays on protoplasts transfected with the *35S::OsRAV11‐4HA* construct were performed. DNA fragments that bound to OsRAV11 were immunoprecipitated using an anti‐HA antibody and used for qRT‐PCR analysis with 5 pairs of PCR primers designed for the amplification of DNA fragments covering the CAACA motif (F1 ~ F5, Figure 4A). Among them, F3 and F4, which are located in the *OsT5H* promoter (1718 bp −1585 bp; 881 bp −739 bp, respectively), showed significant enrichment (Figure 4A). To further identify which CAACA motifs OsRAV11 binds to, Y1H assays with AD‐OsRAV11 as prey and oligonucleotides of different CAACA motif‐containing segments (portion of F1‐F6, Figure 4A) as bait were carried out. To enhance the effect of these Y1H assays, a 20‐bp fragment containing the CAACA motif was synthesised for each site and cloned into a pHIS2 vector with 3 copies of the 20‐bp fragment in tandem; the vectors were designated as pHIS2‐f1 to ‐f6, respectively. Yeast strains co‐expressing pHIS2‐f1, −3 and ‐f4 and AD‐OsRAV11 were able to grow on TDO + 3‐AT medium, but those co‐expressing pHIS2‐f2, −5 and ‐f6 and AD‐OsRAV11 were not (Figure 4B). These results, together with the ChIP‐qPCR results, suggest that OsRAV11 binds to the *OsT5H* promoter through the CAACA motif regions between 1718 bp to 1585 bp and 881 bp to 739 bp.

To investigate the role of OsRAV11 on *OsT5H* transcription in rice plants, two transgene‐free, homozygous *Osrav11* knock‐out mutant lines (*rav11‐1*, *rav11‐2*) were generated by CRISPR/Cas9‐mediated target mutagenesis (Figure 4D; Figure S8). qRT‐PCR analysis showed that the expression levels of *OsT5H* in *rav11‐1* and *rav11‐2* were comparable to those in the wild‐type plants under normal conditions (Figure 4E). However, after 24 h of MD, *OsT5H* transcript abundance in both mutant lines became significantly higher than that in the wild‐type (Figure 4E).

Overexpression lines of *OsRAV11* were produced by expressing *Ubi::OsRAV11‐3Flag* in Jiahua No. 1. Of the 22 lines generated, three lines (#5, #6 and #21) exhibited a moderate increase in *OsRAV11* transcription, with up to 4‐fold increase of transcripts (Figure 4F), and corresponding increase in expression of OsRAV11‐3Flag fusion protein (Figure 4G), compared to the wild‐type. As expected, the *OsT5H* transcript abundance was significantly reduced in all these lines compared to the wild‐type plants (Figure 4H).

To further validate the regulatory effects of OsMYB306 and OsRAV11 on *OsT5H* transcription, dual‐luciferase reporter assays were performed in *N. benthamiana* leaves. Co‐expression of *ProOsT5H‐LUC* with *35S::OsMYB306* or *35S::OsRAV11* markedly reduced luciferase activity compared with the *35S::GFP* control (Figure 4I). When *ProOsT5H‐LUC* was co‐expressed with both *35S::OsMYB306* and *35S::OsRAV11*, the LUC/REN ratio decreased to the lowest level (Figure 4I), indicating that OsMYB306 and OsRAV11 act synergistically to repress *OsT5H* promoter activity.

Taken together, these findings demonstrate that OsRAV11 directly binds to the *OsT5H* promoter and acts together with OsMYB306 to repress its transcription. This cooperative regulation may play an important role in mediating serotonin‐associated defence responses.

### 2.5 Analysis of Expression Patterns of *OsT5H* , *OsMYB306* and *OsRAV11*

To better understand the mechanism by which OsMYB306 and OsRAV11 regulate *OsT5H* transcription in rice, we analysed their spatiotemporal expression patterns using common sets of plant materials.

**FIGURE 5:**
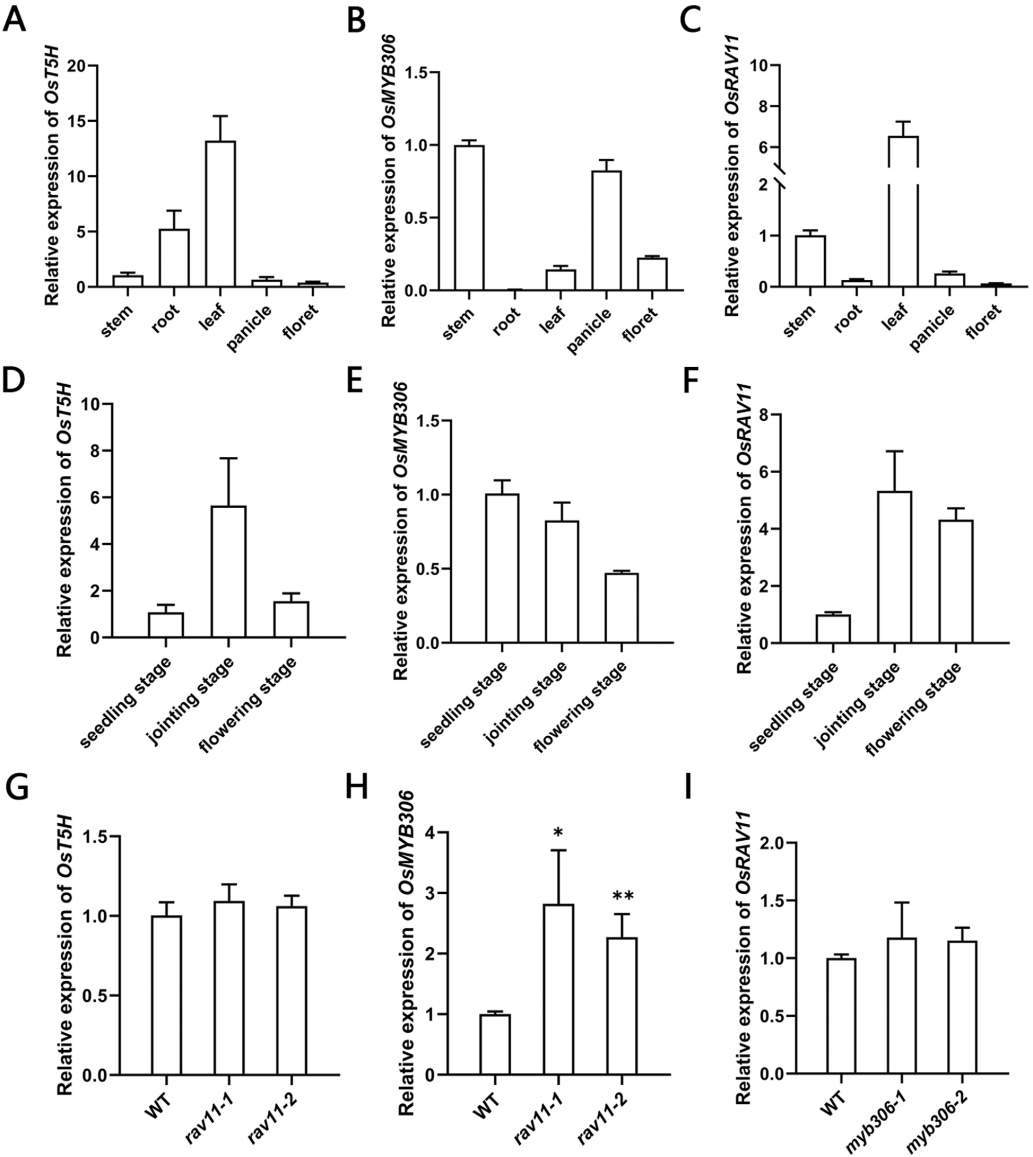
Expression patterns of *OsT5H*, *OsMYB306* and *OsRAV11* in different tissues and developmental stages. (A–C) RT‐qPCR analysis of *OsT5H* (A), *OsMYB306* (B) and *OsRAV11* (C) expression in different tissues of wild‐type rice plants at the flowering stage. Data are shown as mean ± SD of three biological replicates and the expression level in the stem was set to 1. (D–F) RT‐qPCR analysis of *OsT5H* (D), *OsMYB306* (E) and *OsRAV11* (F) expression in leaves at the seedling, jointing and flowering stages in wild‐type rice plants. Data are shown as mean ± SD of three biological replicates and the expression level at the seedling stage was set to 1. (G, H) Relative transcript levels of *OsT5H* (G) and *OsMYB306* (H) in 2‐week‐old wild‐type and *rav11* mutant lines (*rav11‐1*, *rav11‐2*) under normal conditions. Data are shown as mean ± SD of three biological replicates (Student's *t*‐test, *p < 0.05, **p < 0.01). (I) Relative transcript levels of *OsRAV11* in 2‐week‐old wild‐type and *myb306* mutant lines (*myb306‐1*, *myb306‐2*) under normal conditions. Data are shown as mean ± SD of three biological replicates (Student's *t*‐test, *p < 0.05, **p < 0.01).

We first analysed their expression in various tissues of plants at the flowering stage. These results showed that *OsT5H* was expressed strongly in the leaf and root but only weakly in the stem, panicle and floret (Figure 5A). In contrast, *OsMYB306* was predominantly expressed in the stem and panicle, with lower levels in leaf and root (Figure 5B). *OsRAV11* was expressed predominantly in the leaf and mildly in the stem, but barely in root, panicle and floret tissues (Figure 5C).

We next examined their transcript abundance in leaves of wild‐type rice plants at different developmental stages. All three genes were expressed from the seedling to flowering stages but displayed distinct temporal patterns. *OsT5H* expression increased from the seedling to the jointing stage and then declined at the flowering stage (Figure 5D). *OsRAV11* showed a similar pattern, but with the highest transcript level at the jointing stage (Figure 5F). In contrast, *OsMYB306* expression gradually declined over time, with the highest level at the seedling stage and the lowest at flowering (Figure 5E).

We already showed that *OsT5H* expression was significantly upregulated in 2‐week‐old and 4‐week‐old *myb306* seedlings as compared to the WT without MD (Figure 2D,E), whereas no change was observed in the *rav11* mutants (Figures 4E and 5G). When treated with MD, *OsT5H* transcription was significantly increased in both *myb306* and *rav11* mutants as compared to the WT. Since *OsMYB306* and *OsRAV11* act cooperatively to repress *OsT5H*, we further examined the reciprocal effect of mutations on their own expression. The transcript level of *OsMYB306* was significantly elevated in both *rav11‐1* and *rav11‐2* (Figure 5H), while that of *OsRAV11* remained unchanged in the *myb306* lines (Figure 5I), as compared to the WT.

We further examined the relative rapidness of transcriptional response of these three genes to mechanical damage. When the stems of flowering plants were punched, all three genes showed significantly increased transcription within 60 min (Figure S9). Among them, *OsRAV11* showed the quickest response with a significant increase of transcripts within 20 min, followed by *OsT5H* and *OsMYB306*. *OsRAV11* also showed the greatest increase of transcript abundance change (over 4‐fold). The transcript abundance remained mostly stable 90 min post treatment.

### 2.6 Knockout of *OsMYB306* and *OsRAV11* Reduced Resistance of Rice to SSB

The above results clearly demonstrate that knock‐out of either *OsMYB306* or *OsRAV11* increases *OsT5H* transcription after MD treatment, resulting in increased serotonin accumulation. Serotonin is essential for SSB larval growth and development (Wang et al. 2023), thus we hypothesised that the *OsRAV11* and *OsT5H* mutations would affect SSB growth, development and survival. To evaluate their combined effect, a double mutant line (designated *myb306‐2 rav11‐1*) was developed by knocking out *OsRAV11* in the *myb306‐2* line. Insect bioassays were performed by feeding newly hatched SSB larvae rice stems with sheath cut from plants at the flowering stage.

**FIGURE 6:**
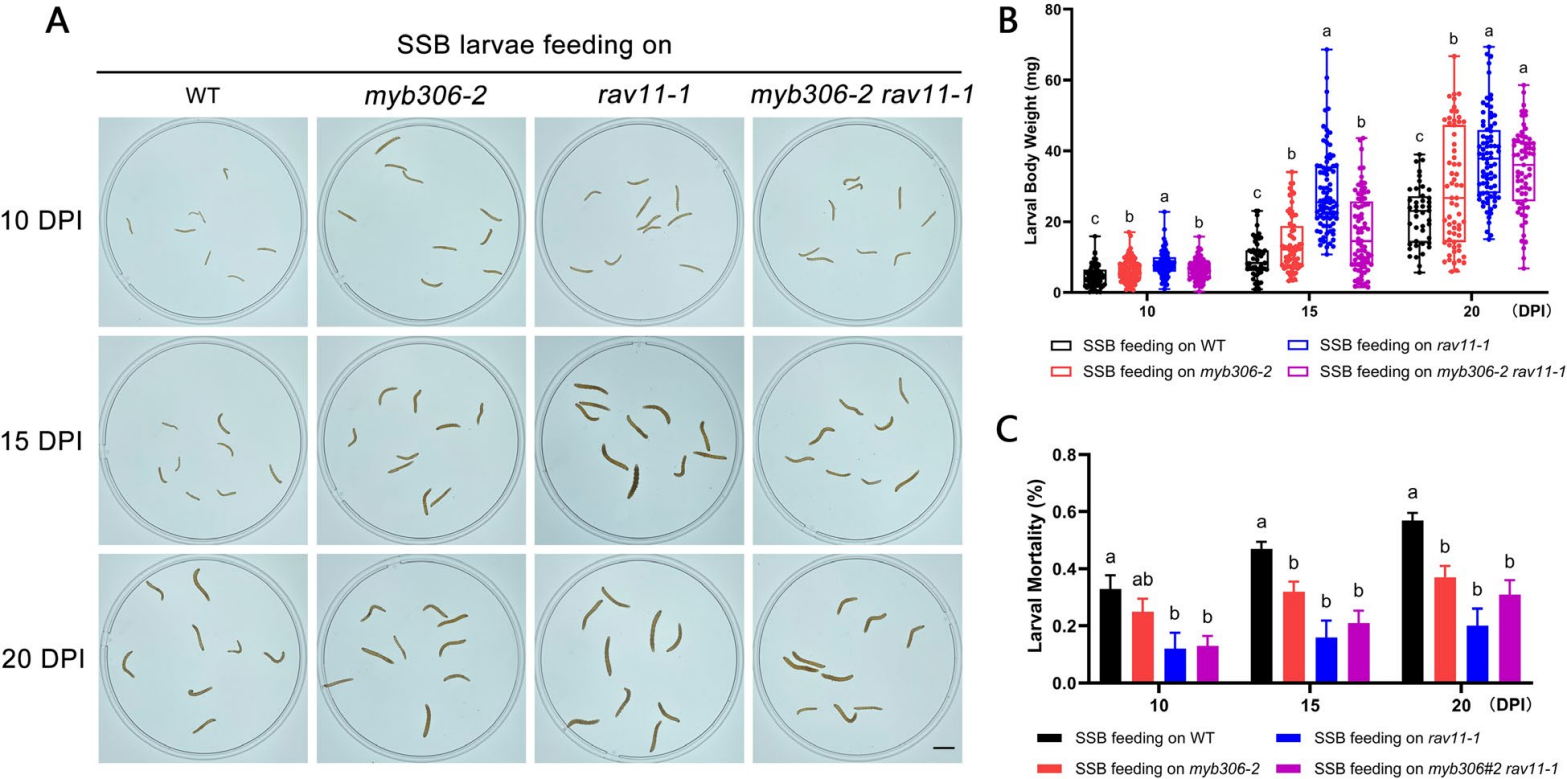
Knockout of *OsMYB306* and *OsRAV11* reduced resistance of rice to SSB. (A) Performance of SSB larvae fed WT, *myb306‐2*, *rav11‐1* or *myb306‐2 rav11‐1* plants at 10, 15 and 20 days post‐infestation. DPI, days post infestation. Scale bars, 1 cm. (B, C) Weight and mortality of SSB larvae fed WT, *myb306‐2*, *rav11‐1* or *myb306‐2 rav11‐1* plants at 10, 15 and 20 days post‐infestation; (B) are shown as boxplots; and (C) are represented by mean ± SEM of 10 biological replicates. Different letters indicate significant difference at *p* < 0.05 level (Student's *t*‐test). DPI, days post infestation.

First, we observed that SBB larvae fed on mutant lines (*myb306‐2*, *rav11‐1* and *myb306‐2 rav11‐1*) grew much better than on the WT and were notably larger 10 days post infestation (DPI) (Figure 6A). Consequently, the body weight of larvae fed on mutant lines was significantly greater than those fed on the WT, with mean body weights (±SD) of 6.26 ± 3.18 mg (10 DPI) to 29.16 ± 16.31 mg (20 DPI) for *myb306‐2*, 7.99 ± 3.48 mg (10 DPI) to 37.97 ± 12.27 mg (20 DPI) for *rav11‐1* and 6.50 ± 2.88 mg (10 DPI) to 34.53 ± 11.56 mg (20 DPI) for *myb306‐2 rav11‐1*, compared with 4.55 ± 3.05 mg (10 DPI) to 21.86 ± 8.31 mg (20 DPI) for the WT (Figure 6B).

Mortality was also recorded over the same time period. The results showed that the mortality rate of larvae fed WT plants increased from 33% (10 DPI) to 57% (20 DPI). This rate was significantly higher than those for larvae fed *myb306‐2* (25% to 37%), *rav11‐1* (12% to 20%), or *myb306‐2 rav11‐1* (13% to 31%) over the same period (Figure 6C). Notably, no significant differences in mortality were observed among the larvae feeding on the three different mutant lines. Furthermore, the increase in larval growth observed on the mutant lines resulted in an increased rate of development, with some of the larvae having already entered the pupal stage 20 days post hatching, compared to none on the WT rice (Figure S10).

The transcript levels of *OsT5H* and 5‐HT contents were further quantified in WT and mutant plants before and after 24 h of SSB feeding. *OsT5H* transcription and 5‐HT accumulation were significantly induced by SSB attack in all genotypes, but their levels were markedly higher in the mutants than in the WT after infestation (Figure S11A,B). The trends observed in *rav11‐1* and *myb306‐2 rav11‐1* were similar and slightly higher than in *myb306‐2*, suggesting that *OsRAV11* may play a predominant role in the cooperative repression module controlling *OsT5H* transcription. These results further confirm that disruption of *OsMYB306* or *OsRAV11* derepresses *OsT5H* transcription, leading to elevated serotonin production.

Together, these results demonstrate that, consistent with the increased serotonin biosynthesis, the knockout of *OsMYB306* and *OsRAV11*, alone or combined, significantly reduced rice resistance to SSB, leading to improved larval growth, survival and development.

### 2.7 Natural Variation and Haplotype Analysis of *OsT5H* , *OsMYB306* and *OsRAV11*

To identify potential functional variants that might be useful in breeding SSB‐resistant rice, we analysed natural variation in the coding regions of *OsMYB306* and *OsRAV11*, as well as the promoter and coding sequences of *OsT5H* in the 3 k rice accessions currently available (Mansueto et al. 2016). No single nucleotide polymorphism (SNP) was detected in *OsMYB306* and only one synonymous SNP (G/A at position 1125 bp, amino acid T375T) was found in *OsRAV11*.

In comparison, there was relatively rich diversity of *OsT5H* across the accessions. In its coding sequence, there was a total of 11 SNPs, including 2 non‐synonymous SNPs (M9V and S32L) and 9 synonymous SNPs. There were also several SNPs in the promoter region; however, none of them are in the binding site of OsMYB306 (−47 bp ~ −38 bp) and OsRAV11 (−1616 bp ~ −1612 bp, −809 bp ~ −805 bp) (Figure S12A).

Based on these SNPs in the *OsT5H* coding region, the 3 k rice population can be classified into 7 major haplotypes, with *japonica* accessions predominantly carrying Hap1 and *indica* varieties displaying higher haplotype diversity (Figure S12B). Similarly, these rice accessions can be divided into 3 major haplotypes based on the SNPs in the promoter region.

## 3 Discussion

With the aim of elucidating the regulatory mechanisms responsible for the induction of serotonin biosynthesis by SSB infestation in rice, this study identified a regulatory module that represses, rather than activates, serotonin biosynthesis. This module consists of two interacting transcription factors, OsMYB306 and OsRAV11, both of which can directly bind to the *OsT5H* promoter, but at different sites. Further, the interaction between OsMYB306 and OsRAV11 cooperatively enhances the repression of *OsT5H* transcription, indicating that the two proteins function as a synergistic repressor complex. These results not only shed light on how to increase the resistance of rice to SSB via regulation of this module, but also on the regulation of the biosynthesis of serotonin, a phytohormone of emerging importance in plants.

Both *OsMYB306* and *OsRAV11* are responsive to SSB infestation, with their encoded proteins binding to the *OsT5H* promoter. This, together with the findings that OsMYB306 and OsRAV11 are interacting transcriptional factors colocalised in the nucleus, strongly suggests they function as a regulatory module in serotonin biosynthesis.

The co‐existence of transcription factors that synergistically regulate gene expression is a conserved strategy in response to variable biotic and abiotic challenges in plants. In rice, MYB22 interacts with the corepressor TOPLESS through its EAR motif and recruits the histone deacetylase HDAC1 to form a MYB22–TOPLESS–HDAC1 complex. This tripartite complex functions as a transcriptional repressor that directly binds to the promoter of *F3′H*, a flavonoid biosynthesis gene negatively associated with rice resistance to the brown planthopper (BPH), thereby repressing its expression and enhancing host defence (Sun et al. 2023). Similarly, under drought stress, OsMYBR57, an MYB‐related transcription factor, interacts with the homeodomain protein OsHB22 to coordinately regulate the expression of drought‐related *OsbZIPs* (Yang et al. 2022). This cooperation ensures precise modulation of stress‐responsive pathways by integrating distinct transcription factor families and their subcellular dynamics.

In our study, SSB infestation activated the transcription of both *OsMYB306* and *OsRAV11*, as well as *OsT5H*. We demonstrated that OsMYB306 and OsRAV11 could jointly repress *OsT5H* expression in vitro and in vivo. Their suppressive effect on *OsT5H* expression was further supported by the fact that, under SSB infestation, *OsT5H* transcript levels were significantly higher in both the *rav11* and *myb306* mutants. The results from spatiotemporal expression analyses also supported the above conclusion: The high expression of *OsMYB306* and *OsRAV11* in stems coincides with the low expression of *OsT5H*. Since SSB mainly feeds on stems in rice, this suppressive OsMYB306‐OsRAV11 module on *OsT5H* expression is likely to play an important role in SSB resistance. Expression of *OsMYB306* and *OsRAV11* was consistently low in roots, where that of *OsT5H* was moderately high. The finding that there was also a high level of *OsT5H* transcripts in leaves, where *OsRAV11* is highly expressed, but *OsMYB306* is not, further suggests that together OsMYB306 and OsRAV11 cause greater suppression on *OsT5H* than either alone.

The knockout effects of *OsMYB306* or *OsRAV11* on *OsT5H* expression were not exactly the same as their differential expression as observed in different tissues. In plants of ~2‐month‐old, *OsT5H* expression was significantly higher in SSB infested *rav11* mutants than in *myb306* mutants (Figure S11A). This finding was not surprising since the expression of *OsMYB306* had already declined in these older plants while *OsRAV11* remained at a high level (Figure 5E), therefore, the mutational effect of the former was expected to be lower than that of the latter. Similarly, it is not unexpected that there was no significant difference in *OsT5H* expression between the double mutant *myb306‐2 rav11‐1* and single *rav11* mutants (Figure S11A). The mutational effect on *OsT5H* expression observed above was ultimately reflected by the concentration of serotonin in rice stem (Figure S11B), i.e., significantly greater serotonin concentrations in both single and double mutant lines of *OsMYB306* or *OsRAV11* than in the WT.

We have previously demonstrated that suppression of serotonin synthesis increases SSB resistance in rice (Lu et al. 2018) and supplementation of serotonin in the diet improves larval growth and survival (Wang et al. 2023). Here, we further demonstrate that an increase of endogenous serotonin in the *OsMYB306* or *OsRAV11* knockout mutant lines improved SSB larval growth (Figure 6A), with significantly increased body weight (Figure 6B) and survival rate (Figure 6C). These results demonstrate a strong correlation between endogenous serotonin levels and SSB resistance in rice. Generation and analysis of double mutants of *OsT5H* and *OsMYB306*/*OsRAV11* may reveal if OsMYB306 or OsRAV11 modulates SSB resistance through mechanisms other than serotonin biosynthesis.

A logical next step is to test *OsMYB306* and *OsRAV11* overexpression lines to explore new strategies for SSB resistance breeding. However, the growth of *OsRAV11* overexpression lines was severely impaired (Figure S13) and their fertility was very low. This finding is not unexpected as *OsRAV11* is a transcriptional factor that, when constitutively overexpressed, often causes pleiotropic negative effects on plant growth. Whether *OsMYB306* overexpression lines exhibit better performance remains to be investigated.

Rice has rich genetic diversity (Nachimuthu et al. 2015). Therefore, another rational way is to explore natural variations for SSB resistance improvement. However, we did not identify any functional diversity of *OsMYB306* and *OsRAV11* based on their coding sequences. We also did not find nucleotide variations in the OsMYB306/OsRAV11 binding sites in the *OsT5H* promoter (Figure S12A). Again, this is not surprising as previous screening of rice genetic collections did not identify any accessions that showed resistance to SSB (Xiang et al. 2023).

As discussed above, the transcription of *OsT5H* and consequently the serotonin level was significantly increased in response to SSB infestation, even though both *OsMYB306* and *OsRAV11* are functioning in the WT plants, which strongly suggests there are other transcriptional factors that induce *OsT5H* expression, as a response to SSB attack. Therefore, further studies are required to identify such regulators for SSB resistance breeding.

In the arms race between pest insects and their host plants, there are often trade‐offs between growth and defence in plants. In this study, the *OsRAV11* overexpression lines had significantly reduced *OsT5H* transcription (Figure 4H) hence are expected to have increased SSB resistance. We observed severe growth defects in these *OsRAV11* overexpression lines (Figure S13); thus, those defects could be a type of trade‐off that negatively affects rice plant growth. However, there seem to be other agonistic transcriptional factors counteracting the effect of OsMYB306‐OsRAV11, hence no clear trade‐off impact was identified in the wild‐type plants.

Because SSB feeding involves both physical (mechanical damage) and biochemical effects (delivery of saliva elicitors) on rice plants, people would like to know whether OsMYB306‐OsRAV11 is activated by the physical or chemical effect, or both. We observed that mechanical damage alone had the same effect on *OsT5H* transcription as SSB feeding and mechanical damage plus saliva treatment (Figure S4), and simple mechanical damage increased the transcription of both *OsMYB306* and *OsRAV11* (Figure S9). A simple understanding of these results would be that physical damage alone could activate the OsMYB306‐OsRAV11 module. Because brown planthopper (BPH) feeding also exerts physical and biochemical effects on rice plants and induces *OsT5H* transcription and serotonin biosynthesis (Lu et al. 2018), we attempted to retrieve public RNA‐seq data, with the aim to examine the response of *OsMYB306* and *OsRAV11* to BPH feeding. However, we failed to identify such data; therefore, further studies are needed to examine whether the OsMYB306‐OsRAV11 module is an SSB responsive model or it is a general model responding to physical damage.

In summary, our present study revealed an SSB‐responsive OsMYB306‐OsRAV11 module that represses the transcription of *OsT5H* and thus plays a positive role in SSB resistance in rice. No functional genetic diversity of this module has been identified in the 3 k collection of rice accessions available to date. Future studies are needed to identify SSB responsive positive regulators of *OsT5H* expression for the development of SSB resistance breeding strategies.

## 4 Experimental Procedures

### 4.1 Plant Materials and Growth Conditions

The *japonica* rice ( *Oryza sativa* L.) cultivar ‘Jiahua #1’ was used as the wild‐type material in this study. The knockout mutants *myb306‐1*, *myb306‐2*, *rav11‐1*, *rav11‐2* as well as *myb306 rav11* double mutants were produced using CRISPR/Cas9‐mediated target mutagenesis. They were developed using two distinct single‐guide RNAs (sgRNAs) to specifically target the first exon of *OsMYB306* (LOC_Os08g33940) and *OsRAV11* (LOC_Os01g49830), respectively (Figures 2C and 5D). For the development of *OsRAV11* overexpressing lines, the full‐length coding sequence of *OsRAV11* was PCR‐amplified and cloned into pENTER‐3FLAG entry vector. Error‐free clones were subsequently introduced into the destination vector pHGW to produce expression vectors by LR recombination reactions (Gateway LR Clonase II, Invitrogen). Transgenic plants were produced via *Agrobacterium*‐mediated transformation (Hiei et al. 1994) of calli induced mature embryos. The primer sequences used to generate the constructs are listed in Table S4.

Field‐grown rice plants were raised under standard paddy conditions in Sanya (Hainan Province, China). For in‐house studies, rice plants were grown hydroponically in Yoshida rice nutrient solution (Yoshida et al. 1976) under controlled environmental conditions in a growth chamber (14 h light/10 h dark, 28°C ± 1°C), with a relative humidity of ~70% ± 10%.

### 4.2 Yeast One‐Hybrid (Y1H) Assay

Y1H assays were performed following the Matchmaker One‐hybrid System (Clontech). For library screening, the promoter sequence of *OsT5H* (2000 bp) was cloned into the pAbAi vector as the bait (pAbAi‐*OsT5H*) and a rice cDNA library was used as the prey. The constructed pAbAi‐*OsT5H* plasmid was linearised by restriction enzyme digestion and integrated into the genome of the Y1HGold yeast strain by homologous recombination to generate a bait‐specific reporter strain Y1HGold (pAbAi‐*OsT5H*). The optimal concentration of Aureobasidin A (AbA) for the bait strain was determined to be 200 ng/mL, which effectively suppressed background growth. The rice cDNA library was transformed into Y1HGold (pAbAi‐*OsT5H*) following the Matchmaker User Manual. Positive clones were selected on synthetic dropout (SD) medium lacking leucine but supplemented with 200 ng/mL AbA. The cDNA inserts from positive clones were sequenced to identify the corresponding genes. For pairwise interaction validation, different *OsT5H* promoter fragments were inserted into the reporter vector pHIS2 and the full‐length cDNA sequences of *OsMYB306* and *OsRAV11* were inserted into the vector pGADT7. Co‐transformation of various combinations was performed according to the manufacturer's instructions. The selection of positive transformants was conducted on SD medium lacking Trp and Leu (DDO). The test of binding between the protein and different promoter fragments was conducted on SD medium lacking Trp, Leu and His (TDO) containing 50 mM 3‐amino‐1,2,4‐triazole (3‐AT). Primers used for vector construction are shown in Table S4.

### 4.3 Electrophoretic Mobility Shift Assay (EMSA)

The coding sequence of *OsMYB306* was cloned into expression vector pET32a. The OsMYB306‐His recombinant protein was induced in *Escherichia coli* BL21 (DE3) using 0.2 mM IPTG (isopropyl β‐D‐1‐thiogalactopyranoside) at 15°C overnight and purified using Ni‐IDA Resin (Novagen). Oligonucleotides were synthesised and labelled with biotin. The unlabeled oligonucleotides were used as competitors. To perform the EMSA, a LightShift Chemiluminescent EMSA kit (Thermo Scientific) was used according to the manufacturer's instructions. Primers and probes used are listed in Table S4.

### 4.4 Dual‐Luciferase Reporter (DLR) Assay

The 2500 bp promoter sequence of *OsT5H* and its derived mutant versions were constructed into the pGreenII 0800‐LUC vector to serve as reporters. Full‐length cDNA sequences of *OsMYB306* and *OsRAV11* were cloned into the pGreenII 62‐SK vector to serve as effectors. To facilitate direct comparison with the GFP control and to assess transcriptional activation or repression under comparable conditions, equal amounts of effector constructs were used in all infiltrations. The effector and reporter pairs were co‐transformed into 3‐week‐old *N. benthamiana* leaves through *Agrobacterium*‐mediated infiltration (Hannah 1996). Activity of firefly luciferase (LUC) and renilla luciferase (REN) was assessed after 48 h using the Dual‐Luciferase Reporter Assay System Kit (Promega). The transcriptional activity was calculated as the ratio of LUC/REN. *35S::GFP* was used as an internal control.

### 4.5 Gene Expression Analysis

For tissue‐specific expression analysis, total RNA was extracted from leaves, stems, panicles, florets and roots of wild‐type rice plants at the flowering stage, using the Plant RNA Kit (Omega). For developmental stage–dependent expression analysis, total RNA was extracted from leaves at the seedling, jointing and flowering stages of wild‐type rice plants.

For MD and SSB mock studies, rice shoot (at tillering stage) or stem (at flowering stage) segments (~2 cm, with the punched site in the middle) were collected for RNA extraction. For short‐term SSB challenge experiments, each rice tiller was infested with a single fourth‐instar SSB larva that had been starved for 2 h prior to infestation. Segments (~2 cm) with an apparent biting hole (where a larva penetrated into rice stem) were cut and used for RNA extraction. The time point when larval mouthparts began penetrating the rice stem (feeding onset) was defined as 0 h, and samples were subsequently collected at 1, 8 and 24 h post feeding onset.

Total RNA was reverse transcribed with Hifair III 1st Strand cDNA Synthesis SuperMix for qPCR (gDNA digester plus) (Yeasen) following the manufacturer's instructions. RT‐qPCR was conducted with Hieff qPCR SYBR Green Master Mix (No Rox) (Yeasen) on a Light Cycler 480 real‐time system (Roche). The relative expression levels were calculated using the comparative 2^−ΔΔCt^ method (Livak and Schmittgen 2001) and normalised to the housekeeping gene *OsActin*. All primers used for gene expression analysis are listed in Table S4.

### 4.6 Subcellular Localisation

The full‐length CDS of *OsMYB306* and *OsRAV11* was amplified and cloned into pGreen‐GFP. For subcellular localisation in rice protoplasts, the recombinant plasmids were transferred into rice protoplasts using the PEG 4000 mediated rice protoplast transformation method (Bart et al. 2006). For subcellular localisation in *N. benthamiana*, the recombinant plasmids were first transformed into *Agrobacterium tumefaciens* and then injected into 3‐week‐old tobacco leaves. Fluorescence signals were observed 48 h post *N. benthamiana* infiltration or 16 h post protoplast transformation using a confocal laser scanning microscope (Olympus FV3000). DAPI (4′,6‐diamino‐2‐phenylindole) was added to visualise the nuclei.

### 4.7 Mechanical Damage and SSB Mock Treatment

For mechanical damage treatment, rice plants were punched in the shoot (at tillering stage) or stem (at flowering stage) using a hole puncher (2 mm in diameter), two punches per shoot/stem.

For SSB feeding mock analysis, SSB saliva was collected from 4‐ or 5‐instar SSB larvae. Selected larvae were first gently rinsed with sterile water, and then lightly stimulated their mouthparts using a micropipette tip to secrete saliva. The saliva was collected with a micropipette and stored at 4°C prior to use within 2 h. A total of 2.5 μL saliva was immediately applied to each punched site.

### 4.8 Quantification of Serotonin Levels in Rice

Serotonin levels in rice were quantified by HPLC as described previously (Kang et al. 2007) with some modifications. For mechanical damage treatments, rice samples were collected from the damaged region of tillers from 4‐week‐old plants at the indicated time point after treatment. For SSB‐related experiments, rice samples were collected from tillers of 2‐month‐old plants, and one ~2.5 cm segment surrounding the bitten site was excised from each infested tiller at the indicated time point after infestation. For the corresponding control groups without MD or SSB treatment, samples were collected from the corresponding positions on untreated tillers. Rice samples were homogenised in liquid nitrogen, and 100 mg tissue was transferred to 2 mL tubes and lysed in 1 mL 80% (v/v) methanol overnight. The homogenates were centrifuged at 14,000×*g* for 10 min at 4°C. The supernatants were filtered (0.45 μm filter) for HPLC analysis (Agilent 1290). Chromatographic separation was carried out on an Agilent Eclipse XDB‐C18 column (150 × 4.6 mm, 5 μm).

### 4.9 Immunoprecipitation‐Mass Spectrometry (IP‐MS) Assay

Total proteins were extracted from rice tissues, collected from plants including SSB infested plants (24 h post infestation), using Cell lysis buffer for Western and IP (Beyotime). Protein extracts were then incubated with OsMYB306‐His recombinant protein and Anti‐His magnetic beads (Biolinkedin) at 4°C for 4 h to facilitate protein binding according to the manufacturer's instructions; protein extracts incubated only with Anti‐His magnetic beads served as a blank control. Mass spectrometry (MS) analysis of the IP products was carried out by QLbio (Beijing, China).

### 4.10 Yeast Two‐Hybrid (Y2H) Assay

For Y2H assays, the coding sequences of *OsRAV11* and *OsMYB306* or its truncated forms were amplified and cloned into pGADT7 and pGBKT7, respectively. Y2H assays were carried out following the Yeastmaker Yeast Transformation System 2 (Clontech). Primers used for vector construction are listed in Table S4.

### 4.11 Bimolecular Fluorescence Complementation (BiFC) Assays

The full‐length coding sequences of *OsMYB306* and *OsRAV11* were cloned into pGreen‐based binary vectors to generate OsMYB306‐cEYFP and nEYFP‐OsRAV11 fusion constructs, respectively. The plasmids were transformed into *Agrobacterium* and then different combinations were co‐infiltrated into the epidermal cells of 3‐week‐old *N. benthamiana* leaves. The fluorescence signals were observed after 48 h incubation using a confocal laser scanning microscope (Olympus FV3000). DAPI (4′,6‐diamino‐2‐phenylindole) served as a nuclear marker.

### 4.12 Luciferase Complementation Imaging Assay (LCI)

Full‐length coding sequences of *OsMYB306* and *OsRAV11* were fused with the N‐terminus of luciferase or C‐termini of luciferase, respectively (OsMYB306‐nLUC and cLUC‐OsRAV11/OsRAV11‐nLUC and cLUC‐OsMYB306). The constructs were separately transformed into *Agrobacterium*. *Agrobacterium* with different combinations of vectors was co‐infiltrated into 3‐week‐old *N. benthamiana* leaves. After 48 h cultivation, signals were observed on a TANON Chemiluminescent Imaging system (Tanon 5200).

### 4.13 Co‐Immunoprecipitation (Co‐IP) Assay

For the Co‐IP assay, the constructs *Ubi::OsMYB306‐4HA* and *Ubi::OsRAV11‐3Flag* were generated and co‐transformed into rice protoplasts. After 16 h incubation in the dark, total proteins were extracted and subsequently incubated with anti‐FLAG magnetic beads (Sigma) at 4°C for 4 h. Both the total protein extracts (used as inputs) and the proteins immunoprecipitated by magnetic beads were detected by Western blot using anti‐HA (Sigma) and anti‐FLAG antibodies (Sigma).

### 4.14 Chromatin Immunoprecipitation Quantitative PCR (ChIP‐qPCR) Assay

ChIP–qPCR assay was performed as described (Wei et al. 2022) with modifications. Briefly, the construct *Ubi::OsRAV11‐3Flag* was transformed into rice protoplasts using the PEG 4000 mediated rice protoplast transformation method (Bart et al. 2006) and incubated for 16 h in the dark. Samples were fixed in 1% (v/v) formaldehyde for 10 min and quenched with 5 mM glycine. The nuclear extract was isolated and sonicated to obtain DNA fragments of 300–500 bp using a Covaris M220 (Covaris). Fragments were precleared with Dynabeads protein A/G (Invitrogen) for 1 h before incubation for 6 h with anti‐Flag magnetic beads (Sigma). The immunoprecipitated samples were used for qPCR analysis. The primers used for ChIP‐PCR are listed in Table S4.

### 4.15 SSB Bioassays

SSB artificial infestation was performed as described (Wang et al. 2023). In brief, rice tillers were detached from 2‐month‐old plants cultivated under standard paddy field conditions in Sanya. Tillers were placed into transparent glass test tubes (height 30 cm, diameter 3 cm), one tiller per tube with ~10 mL Yoshida rice nutrient solution.

For SSB performance assays assessing larval growth, survival and development, 10 newly hatched SSB larvae were transferred to one tiller. Ten days postinfestation (DPI), larval mortality was recorded and the individual weights of surviving larvae were measured; these were then individually transferred to a new tube containing a new, healthy rice tiller. This experiment was also performed at 15 DPI and 20 DPI with 10 biological replicates.

### 4.16 Statistical Analysis

Student's *t*‐test (*p* < 0.05) was used for significant difference analysis between two samples. One‐way analysis of variance (ANOVA) followed by Tukey's test (*p* < 0.05) was used for multiple comparisons. All the analyses were performed using GraphPad Prism (Version 8).

## Author Contributions

Q.‐Y.S. planned and designed the research. J.‐R.Z., L.W. and Y.‐F.C. performed the research. X.‐H.G. contributed the bioinformatic analyses. Q.W. and Q.Q. generated the mutant lines. J.‐R.Z., M.J., Y.‐Y.T., A.M.R.G. and Q.‐Y.S. analysed the data and wrote the paper. All authors reviewed and approved the final manuscript.

## Funding

This work was supported by the National Natural Science Foundation of China (No. 32072034) and in part by the Hainan Provincial Department of Science and Technology (ZDYF2025XDNY093).

## Conflicts of Interest

The authors declare no conflicts of interest.

## Supporting information


**Figure S1:** Y1H analysis confirms a positive interaction between *OsT5H* promoter and TFs outlined in Table S2.
**Figure S2:** Subcellular localisation of OsMYB306.
**Figure S3:** Structural and sequence alignment of *OsMYB306* and its mutant forms.
**Figure S4:** Relative expression of *OsT5H* in rice subjected to mechanical damage and SSB feeding.
**Figure S5:** FPKM values of transcription factors in response to SSB infestation.
**Figure S6:** Homology alignment of AtRAV1 and OsRAV11.
**Figure S7:** Subcellular localisation of OsRAV11.
**Figure S8:** Structural and sequence alignment of *OsRAV11* and its mutant forms.
**Figure S9:** Temporal dynamics of *OsT5H*, *OsMYB306* and *OsRAV11* in response to mechanical damage.
**Figure S10:** Performance of SSB larvae fed on different materials.
**Figure S11:** Relative expression of *OsT5H* and 5‐HT content in the WT and mutant plants after SSB feeding.
**Figure S12:** Natural variation and haplotype analysis of *OsT5H* in the 3 k rice collection.
**Figure S13:** Growth phenotypes of WT and *OsRAV11* overexpression lines.

**Table S1:** Genes revealed by sequencing of positive clones in a Y1H library screen using *OsT5H* promoter as bait.
**Table S2:** Transcription factors that could bind to the *OsT5H* promoter, as predicted by PlantRegMap.
**Table S3:** Potential transcription factors interacting with OsMYB306 identified by IP‐MS analysis.
**Table S4:** Primers used in this study.

## Data Availability

All the data generated or analysed during this study are presented in the manuscript.

## References

[pbi70680-bib-0001] Bart, R. , M. Chern , C.‐J. Park , L. Bartley , and P. C. Ronald . 2006. “A Novel System for Gene Silencing Using siRNAs in Rice Leaf and Stem‐Derived Protoplasts.” Plant Methods 2, no. 1: 13. 16808845 10.1186/1746-4811-2-13 PMC1524957

[pbi70680-bib-0002] Chen, C. , Y. Li , H. Zhang , et al. 2021. “Genome‐Wide Analysis of the RAV Transcription Factor Genes in Rice Reveals Their Response Patterns to Hormones and Virus Infection.” Viruses 13, no. 5: 752. 33922971 10.3390/v13050752 PMC8146320

[pbi70680-bib-0003] Du, B. , W. Zhang , B. Liu , et al. 2009. “Identification and Characterization of Bph14, a Gene Conferring Resistance to Brown Planthopper in Rice.” Proceedings of the National Academy of Sciences of the United States of America 106, no. 52: 22163–22168. 20018701 10.1073/pnas.0912139106 PMC2793316

[pbi70680-bib-0004] Erland, L. A. E. , S. J. Murch , R. J. Reiter , and P. K. Saxena . 2015. “A New Balancing Act: The Many Roles of Melatonin and Serotonin in Plant Growth and Development.” Plant Signaling & Behavior 10, no. 11: e1096469. 26418957 10.1080/15592324.2015.1096469 PMC4883872

[pbi70680-bib-0005] Fahad, S. , L. Nie , S. Hussain , et al. 2015. “Rice Pest Management and Biological Control.” In Sustainable Agriculture Reviews: Cereals, edited by E. Lichtfouse and A. Goyal , 85–106. Springer International Publishing.

[pbi70680-bib-0006] Fujiwara, T. , S. Maisonneuve , M. Isshiki , et al. 2010. “Sekiguchi Lesion Gene Encodes a Cytochrome P450 Monooxygenase That Catalyzes Conversion of Tryptamine to Serotonin in Rice.” Journal of Biological Chemistry 285, no. 15: 11308–11313. 20150424 10.1074/jbc.M109.091371 PMC2857009

[pbi70680-bib-0007] Hannah, R. 1996. “Dual‐Luciferase TM Reporter Assay: An Advanced Co‐Reporter Technology Integrating Firefly and Renilla Luciferase Assays.”

[pbi70680-bib-0008] Hiei, Y. , S. Ohta , T. Komari , and T. Kumashiro . 1994. “Efficient Transformation of Rice ( *Oryza sativa* L.) Mediated by Agrobacterium and Sequence Analysis of the Boundaries of the T‐DNA.” Plant Journal 6, no. 2: 271–282. 10.1046/j.1365-313x.1994.6020271.x 7920717

[pbi70680-bib-0009] Ikeda, M. , and M. Ohme‐Takagi . 2009. “A Novel Group of Transcriptional Repressors in Arabidopsis.” Plant & Cell Physiology 50, no. 5: 970–975. 19324928 10.1093/pcp/pcp048

[pbi70680-bib-0010] Ishihara, A. , Y. Hashimoto , H. Miyagawa , and K. Wakasa . 2008. “Induction of Serotonin Accumulation by Feeding of Rice Striped Stem Borer in Rice Leaves.” Plant Signaling & Behavior 3, no. 9: 714–716. 19704837 10.4161/psb.3.9.6456 PMC2634568

[pbi70680-bib-0011] Kagaya, Y. , K. Ohmiya , and T. Hattori . 1999. “RAV1, a Novel DNA‐Binding Protein, Binds to Bipartite Recognition Sequence Through Two Distinct DNA‐Binding Domains Uniquely Found in Higher Plants.” Nucleic Acids Research 27, no. 2: 470–478. 9862967 10.1093/nar/27.2.470 PMC148202

[pbi70680-bib-0012] Kang, S. , K. Kang , K. Lee , and K. Back . 2007. “Characterization of Tryptamine 5‐Hydroxylase and Serotonin Synthesis in Rice Plants.” Plant Cell Reports 26, no. 11: 2009–2015. 17639402 10.1007/s00299-007-0405-9

[pbi70680-bib-0013] Katiyar, A. , S. Smita , S. K. Lenka , R. Rajwanshi , V. Chinnusamy , and K. C. Bansal . 2012. “Genome‐Wide Classification and Expression Analysis of MYB Transcription Factor Families in Rice and Arabidopsis.” BMC Genomics 13: 544. 23050870 10.1186/1471-2164-13-544 PMC3542171

[pbi70680-bib-0014] Livak, K. J. , and T. D. Schmittgen . 2001. “Analysis of Relative Gene Expression Data Using Real‐Time Quantitative PCR and the 2−ΔΔCT Method.” Methods 25, no. 4: 402–408. 11846609 10.1006/meth.2001.1262

[pbi70680-bib-0015] Lu, H. , T. Luo , H. Fu , et al. 2018. “Resistance of Rice to Insect Pests Mediated by Suppression of Serotonin Biosynthesis.” Nature Plants 4, no. 6: 338–344. 29735983 10.1038/s41477-018-0152-7

[pbi70680-bib-0016] Mansueto, L. , R. R. Fuentes , D. Chebotarov , et al. 2016. “SNP‐Seek II: A Resource for Allele Mining and Analysis of Big Genomic Data in *Oryza sativa* .” Current Plant Biology 7–8: 16–25.

[pbi70680-bib-0017] Nachimuthu, V. V. , R. Muthurajan , S. Duraialaguraja , et al. 2015. “Analysis of Population Structure and Genetic Diversity in Rice Germplasm Using SSR Markers: An Initiative Towards Association Mapping of Agronomic Traits in *Oryza sativa* .” Rice 8, no. 1: 30. 26407693 10.1186/s12284-015-0062-5 PMC4583558

[pbi70680-bib-0018] Osnato, M. , L. Matias‐Hernandez , A. E. Aguilar‐Jaramillo , M. M. Kater , and S. Pelaz . 2020. “Genes of the RAV Family Control Heading Date and Carpel Development in Rice.” Plant Physiology 183, no. 4: 1663–1680. 32554473 10.1104/pp.20.00562 PMC7401134

[pbi70680-bib-0019] Savary, S. , L. Willocquet , S. J. Pethybridge , P. Esker , N. McRoberts , and A. Nelson . 2019. “The Global Burden of Pathogens and Pests on Major Food Crops.” Nature Ecology & Evolution 3, no. 3: 430–439. 30718852 10.1038/s41559-018-0793-y

[pbi70680-bib-0020] Shu, Q. , G. Ye , H. Cui , et al. 2000. “Transgenic Rice Plants With a Synthetic cry1Ab Gene From *Bacillus thuringiensis* Were Highly Resistant to Eight Lepidopteran Rice Pest Species.” Molecular Breeding 6, no. 4: 433–439.

[pbi70680-bib-0021] Sun, B. , Y. Shen , S. Chen , Z. Shi , H. Li , and X. Miao . 2023. “A Novel Transcriptional Repressor Complex MYB22–TOPLESS–HDAC1 Promotes Rice Resistance to Brown Planthopper by Repressing F3′H Expression.” New Phytologist 239, no. 2: 720–738. 37149887 10.1111/nph.18958

[pbi70680-bib-0022] Sun, L. , J. Yin , L. Wang , et al. 2025. “Role of Serotonin in Plant Stress Responses: Quo Vadis? JIPB.” Journal of Integrative Plant Biology 67, no. 7: 1706–1724. 40098453 10.1111/jipb.13882

[pbi70680-bib-0023] Sun, Y. , B. Wang , J. Ren , et al. 2022. “OsbZIP18, a Positive Regulator of Serotonin Biosynthesis, Negatively Controls the UV‐B Tolerance in Rice.” International Journal of Molecular Sciences 23, no. 6: 3215. 35328636 10.3390/ijms23063215 PMC8949417

[pbi70680-bib-0024] Thapa, R. , and N. Bhusal . 2020. “Designing Rice for the 22nd Century: Towards a Rice With an Enhanced Productivity and Efficient Photosynthetic Pathway.” Turkish Journal of Agriculture ‐ Food Science and Technology 8, no. 12: 2623–2634.

[pbi70680-bib-0025] Wang, L. , H. Lu , X. Zhang , et al. 2023. “Disruption of Serotonin Biosynthesis Increases Resistance to Striped Stem Borer Without Changing Innate Defense Response in Rice.” Journal of Pineal Research 75, no. 2: e12895. 37392131 10.1111/jpi.12895

[pbi70680-bib-0026] Wei, H. , H. Xu , C. Su , X. Wang , and L. Wang . 2022. “Rice CIRCADIAN CLOCK ASSOCIATED 1 Transcriptionally Regulates ABA Signaling to Confer Multiple Abiotic Stress Tolerance.” Plant Physiology 190, no. 2: 1057–1073. 35512208 10.1093/plphys/kiac196 PMC9516778

[pbi70680-bib-0027] Wei, Y. , Y. Chang , H. Zeng , G. Liu , C. He , and H. Shi . 2018. “RAV Transcription Factors Are Essential for Disease Resistance Against Cassava Bacterial Blight via Activation of Melatonin Biosynthesis Genes.” Journal of Pineal Research 64, no. 1: e12454. 10.1111/jpi.12454 29151275

[pbi70680-bib-0028] Wei, Y. , H. Xie , L. Xu , et al. 2024. “Coat Protein of Cassava Common Mosaic Virus Targets RAV1 and RAV2 Transcription Factors to Subvert Immunity in Cassava.” Plant Physiology 194, no. 2: 1218–1232. 37874769 10.1093/plphys/kiad569

[pbi70680-bib-0029] Wei, Y. , H. Zeng , W. Hu , L. Chen , C. He , and H. Shi . 2016. “Comparative Transcriptional Profiling of Melatonin Synthesis and Catabolic Genes Indicates the Possible Role of Melatonin in Developmental and Stress Responses in Rice.” Frontiers in Plant Science 7: 676. 27242875 10.3389/fpls.2016.00676 PMC4870392

[pbi70680-bib-0030] Xiang, X. , S. Liu , H. Li , A. D. Ofori , X. Yi , and A. Zheng . 2023. “Defense Strategies of Rice in Response to the Attack of the Herbivorous Insect, Chilo Suppressalis.” International Journal of Molecular Sciences 24: 14361. 37762665 10.3390/ijms241814361 PMC10531896

[pbi70680-bib-0031] Yang, L. , Y. Chen , L. Xu , et al. 2022. “The OsFTIP6‐OsHB22‐OsMYBR57 Module Regulates Drought Response in Rice.” Molecular Plant 15, no. 7: 1227–1242. 35684964 10.1016/j.molp.2022.06.003

[pbi70680-bib-0032] Yoshida, S. , D. A. Forno , J. H. Cock , and K. A. Gomez . 1976. “Laboratory Manual for Physiological Studies of Rice.”

