# Supplementary material for: OsMYB306‐OsRAV11 Regulates Resistance of Rice to Striped Stem Borer by Modulating Serotonin Biosynthesis
Source: Plant Biotechnol J. 2026 May 7;24(8):4951–65. doi: 10.1111/pbi.70680 (PMC13387888; doi:10.1111/pbi.70680)
Supplement: Supplementary file 1 — Figure S1: Y1H analysis confirms a positive interaction between OsT5H promoter and TFs outlined in Table S2. Figure S2: Subcellular localisation of OsMYB306. Figure S3: Structural and sequence alignment of OsMYB306 and its mutant forms. Figure S4: Relative expression of OsT5H in rice subjected to mechanical damage and SSB feeding. Figure S5: FPKM values of transcription factors in response to SSB infestation. Figure S6: Homology alignment of AtRAV1 and OsRAV11. Figure S7: Subcellular localisation of OsRAV11. Figure S8: Structural and sequence alignment of OsRAV11 and its mutant forms. Figure S9: Temporal dynamics of OsT5H, OsMYB306 and OsRAV11 in response to mechanical damage. Figure S10: Performance of SSB larvae fed on different materials. Figure S11: Relative expression of OsT5H and 5‐HT content in the WT and mutant plants after SSB feeding. Figure S12: Natural variation and haplotype analysis of OsT5H in the 3 k rice collection. Figure S13: Growth phenotypes of WT and OsRAV11 overexpression lines. [file PBI-24-4951-s001.docx]

**Supplemental figures for the manuscript**

**OsMYB306-OsRAV11 regulates resistance of rice to** **striped stem borer by modulating serotonin biosynthesis**

**
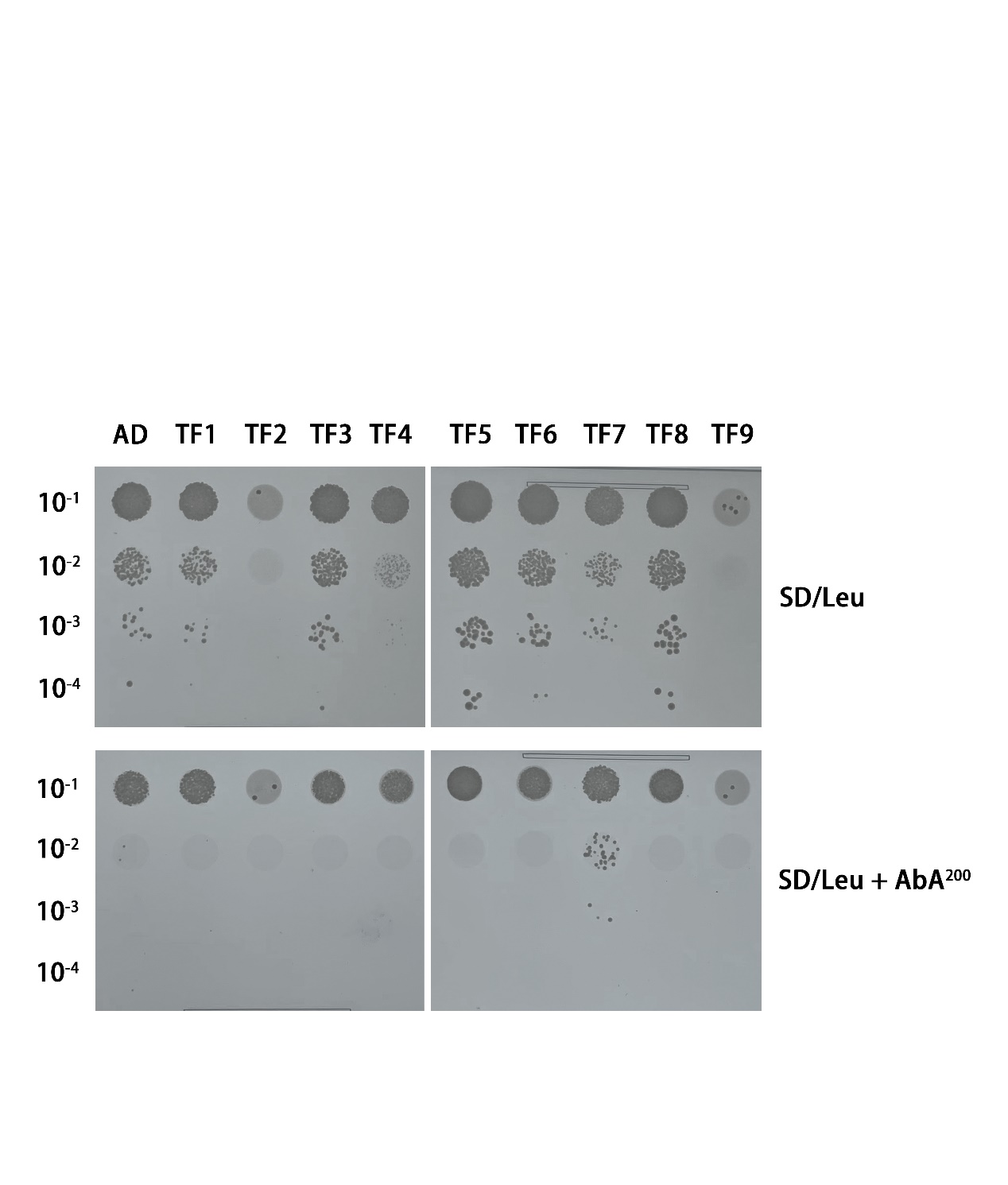
Supplemental Figure 1. Y1H analysis confirms a positive interaction between *OsT5H* promoter and TFs outlined in Supplementary Table 2.**

Transcription factors TF1~TF9 were identified based on results from Y1H library screening (Supplemental Table 2). Transformed yeast strains were grown on synthetic dropout medium lacking leucine (SD/-Leu) and tested for AbA resistance on SD/-Leu but supplemented with 200 ng/ml AbA. Empty vector (AD) served as a negative control.


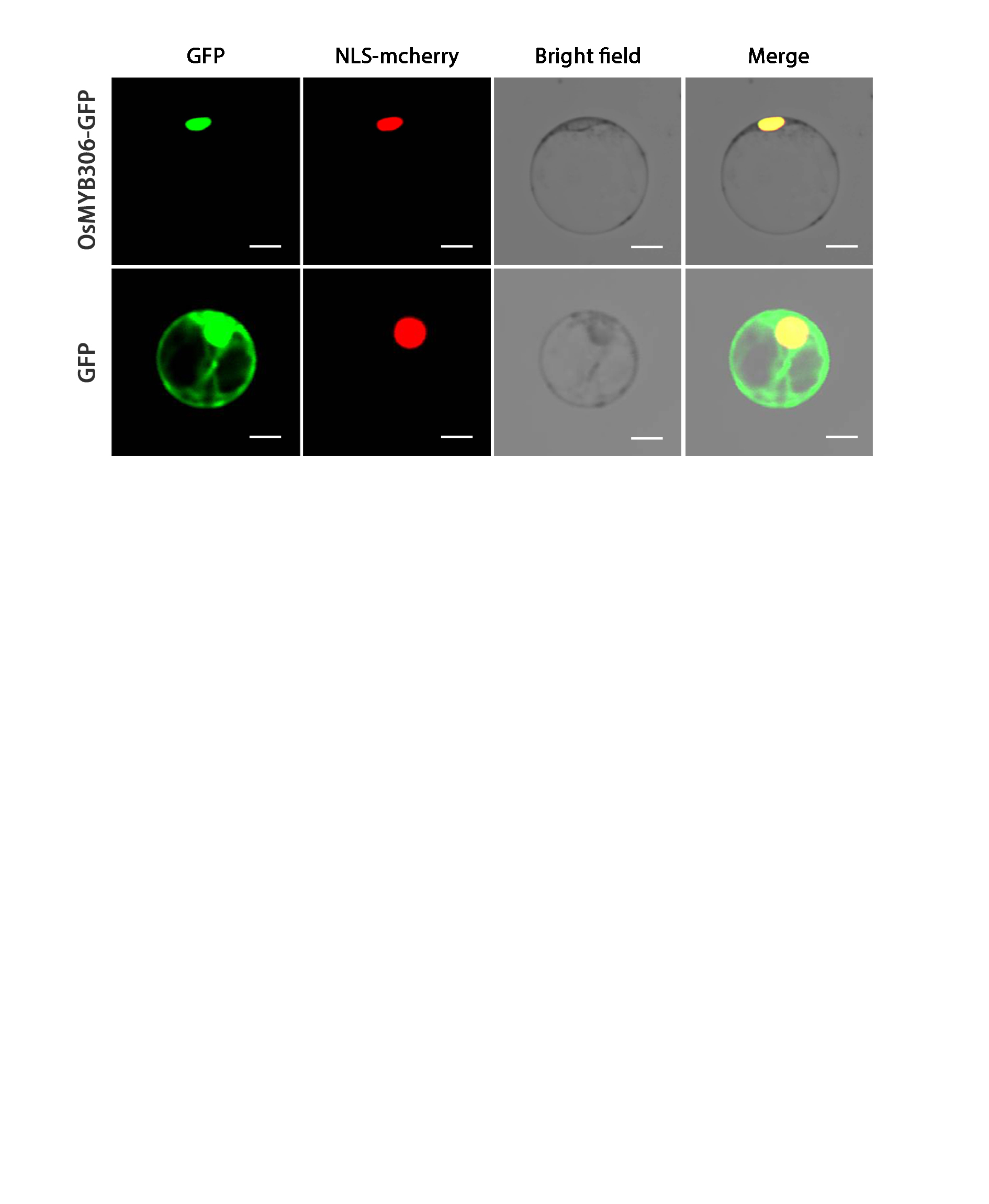
**Supplemental Figure 2. Subcellular localization of OsMYB306.**

Subcellular localization of OsMYB306-GFP in rice protoplasts. Free GFP was used as a control. GFP, GFP fluorescence; NLS-mCherry, red fluorescence of a nuclear marker; Merge, merged GFP, NLS-mCherry, and bright field images. Scale bars, 10 μm.


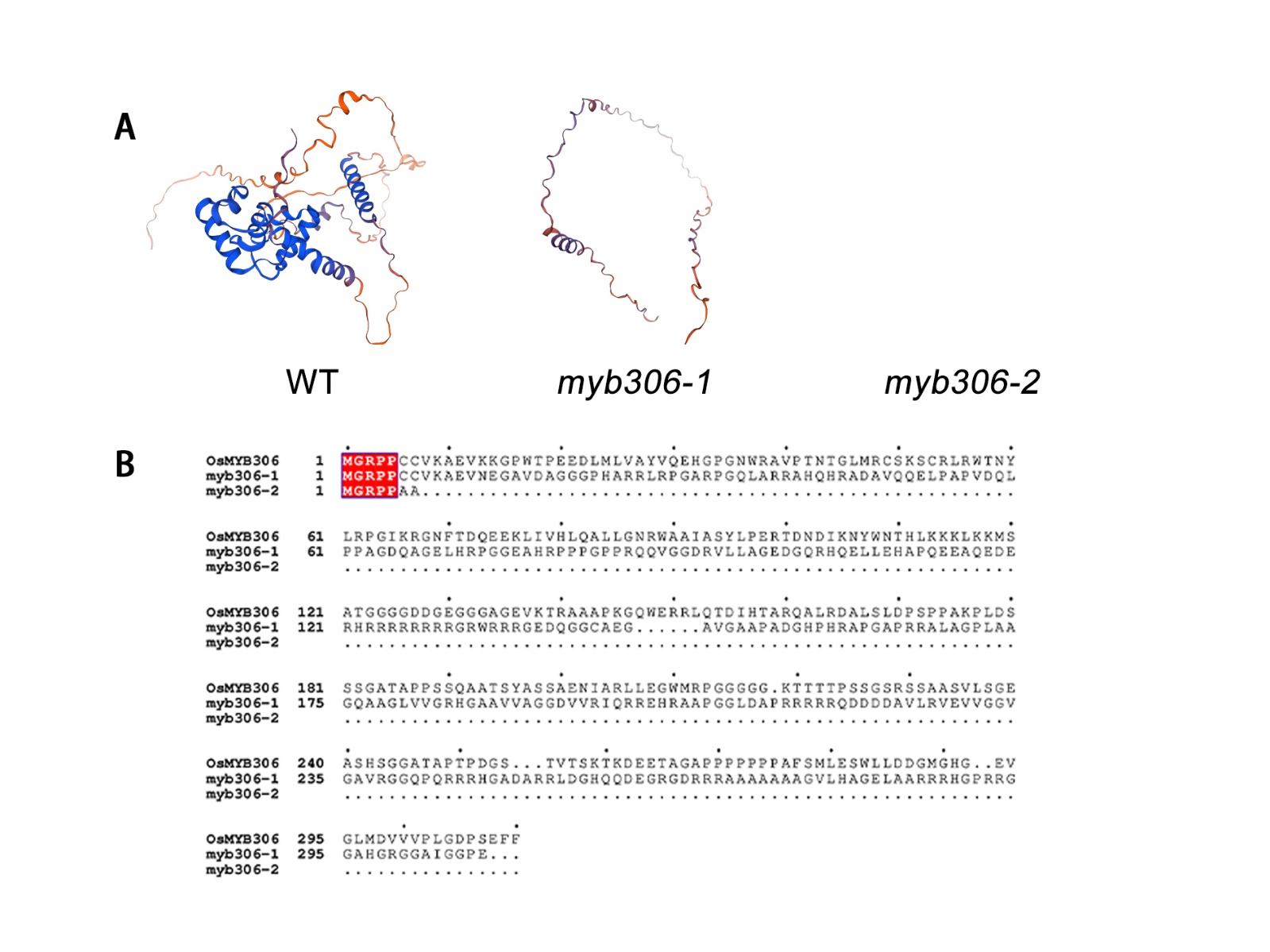
**Supplemental Figure 3. Structural and sequence alignment of *OsMYB306* and its mutant forms.**

(A) The three-dimensional structures of *OsMYB306* and its mutant forms were predicted using SWISSMODEL (https://www.swissmodel.expasy.org/).

(B) Amino acid sequence alignment between the wild-type and mutant proteins was performed using ESPript 3.0 (https://espript.ibcp.fr/ESPript/cgi-bin/ESPript.cgi)


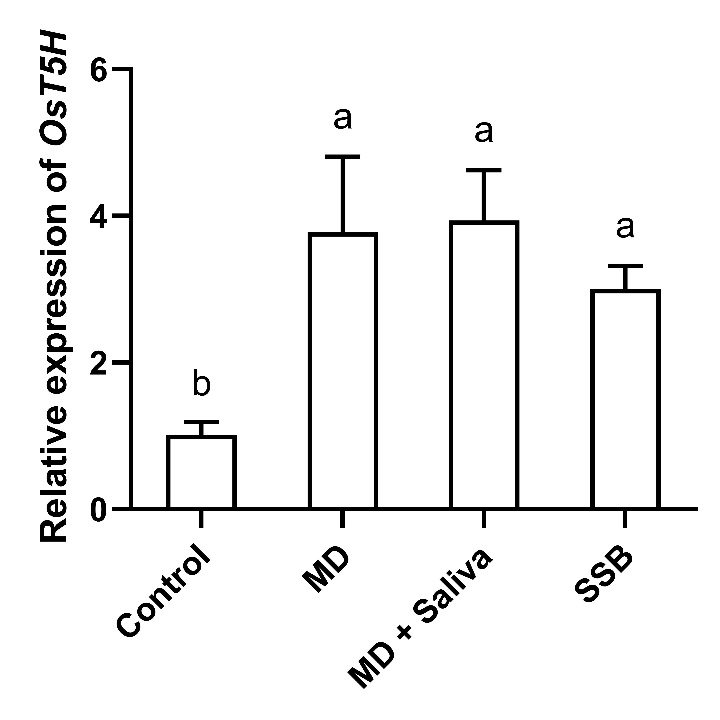
**Supplemental Figure 4. Relative expression of *OsT5H* in rice subjected to mechanical damage and SSB feeding.**

Wild-type rice plants at the flowering stage were treated with MD, MD combined with SSB saliva (MD + SSB saliva) or SSB feeding. For the MD + saliva treatment, 2.5 μL SSB saliva was immediately applied to mechanically wounded sites. *OsT5H* expression levels were measured 3 h post-treatment using RT-qPCR. Data are presented as relative expression levels normalized to the control (untreated plants). Statistical analysis by one-way ANOVA. Different letters indicate significant difference at P < 0.05 level. Error bars indicate SD. MD, mechanical damage; SSB, striped stem borer.


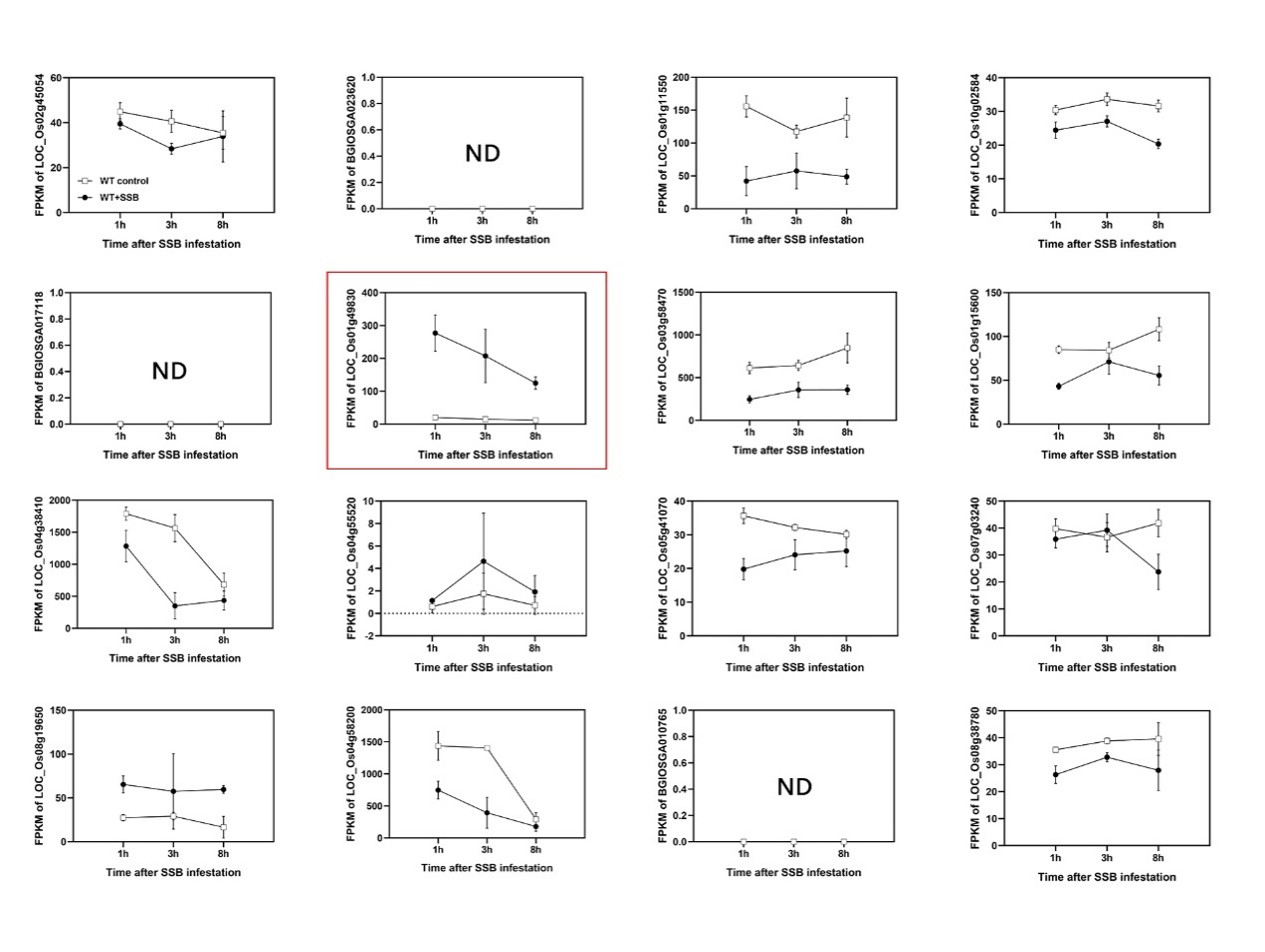
**Supplemental Figure 5. FPKM values of transcription factors in response to SSB infestation.**

RNA-seq data analysis of 16 transcription factors, as detailed in Supplemental Table 3, at 1, 3, and 8 hours post-SSB infestation compared to control plants without infestation. Data points represent the average FPKM values for each transcription factor across three biological replicates. ND, not detected.


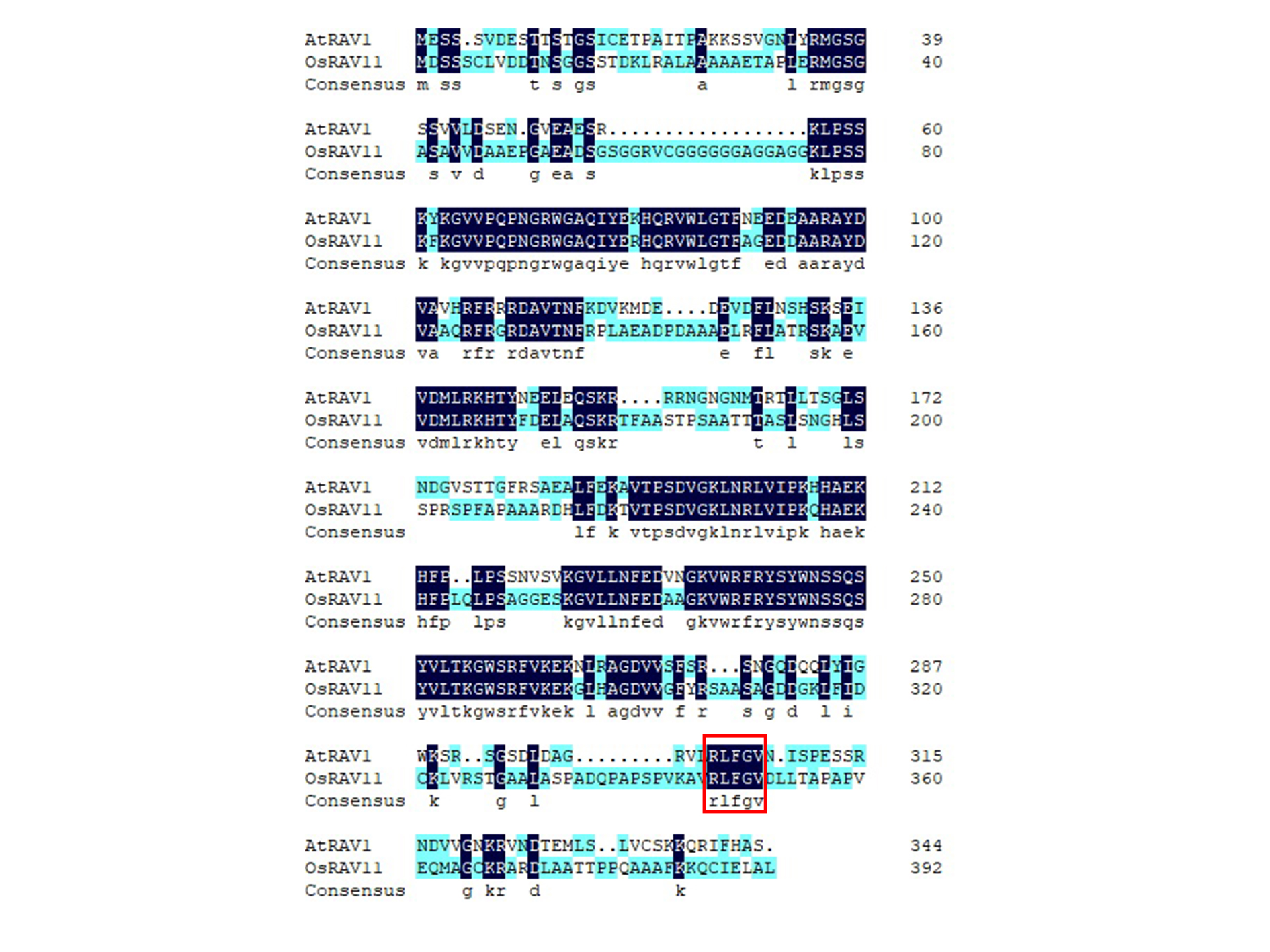
**Supplemental Figure 6.** **Homology alignment of AtRAV1 and OsRAV11.**

Protein sequence alignment of AtRAV1 and OsRAV11 using DNAMAN software. The alignment highlights the conserved C-terminal repression domain RLFGV (boxed in red), which is shared between the two proteins.


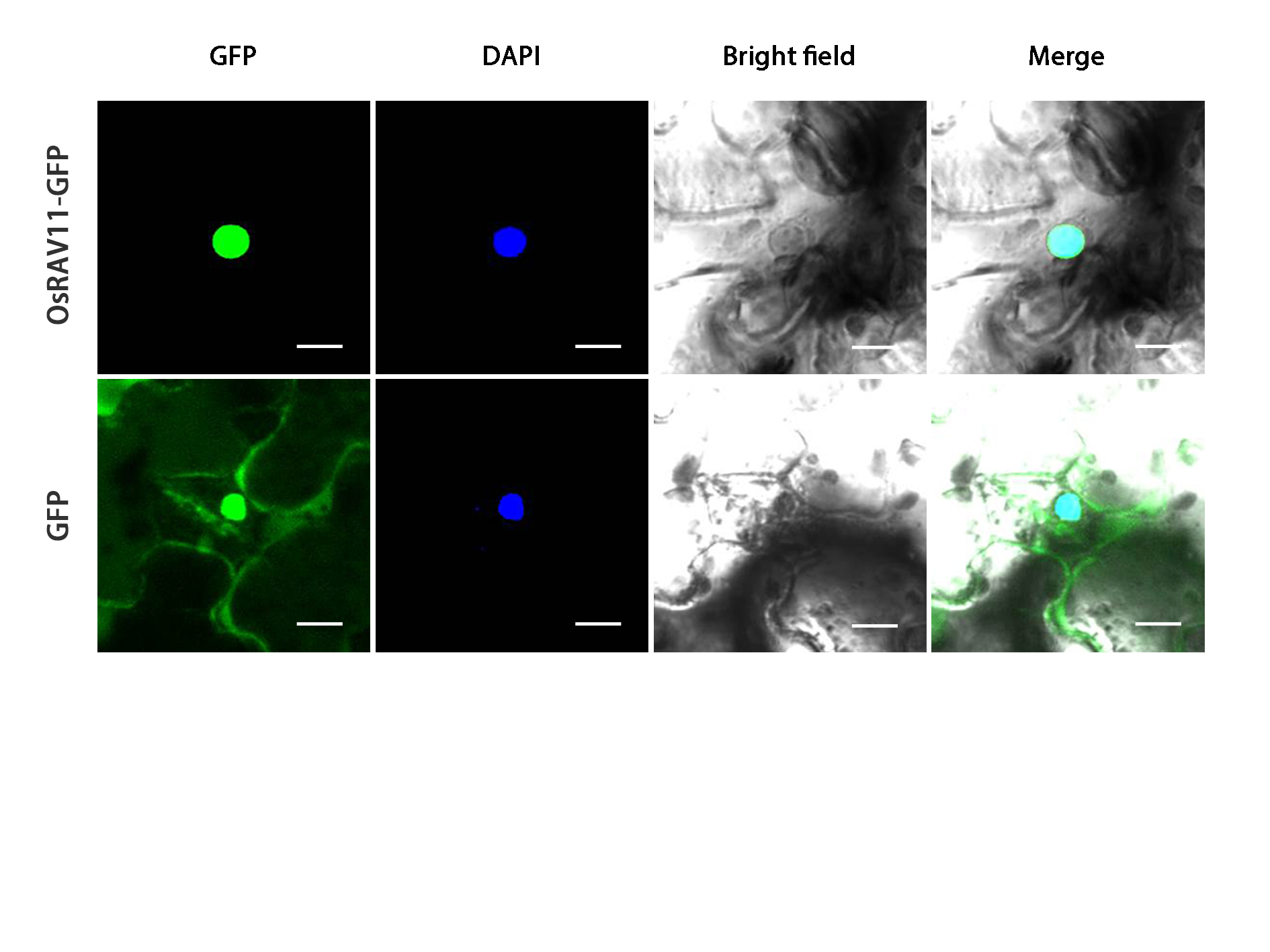
**Supplemental Figure 7.** **Subcellular localization of OsRAV11.**

Subcellular localization of OsRAV11-GFP in *N. benthamiana* leaf epidermal cells. GFP, GFP fluorescence; DAPI, fluorescence of 4’,6-diamino-2-phenylindole; Merge, merged GFP, DAPI, and bright field images. Scale bar, 10 μm.


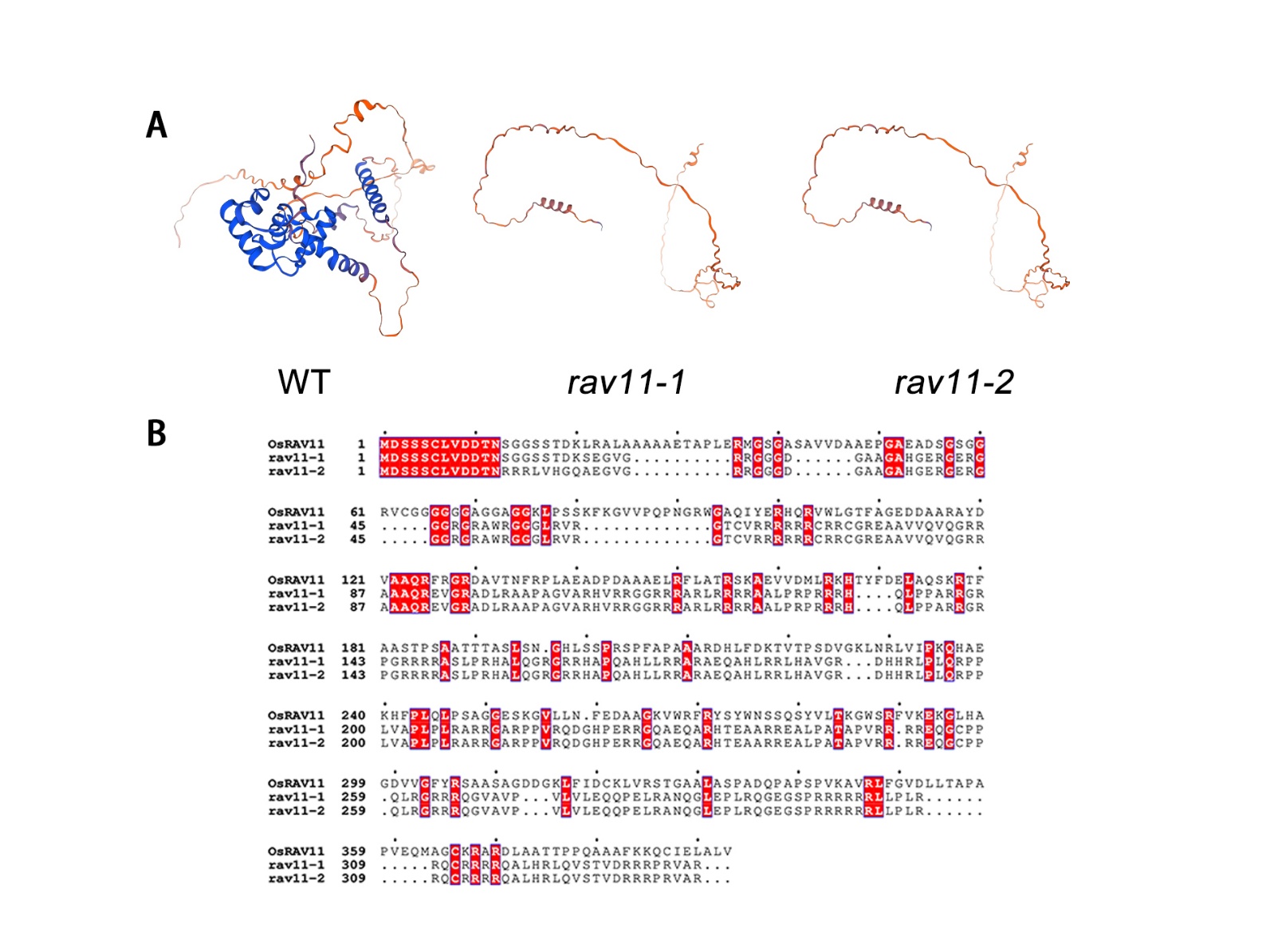
**Supplemental Figure 8. Structural and sequence alignment of *OsRAV11* and its mutant forms.**

(A) The three-dimensional structures of *OsRAV11* and its mutant forms were predicted using SWISSMODEL (https://www.swissmodel.expasy.org/).

(B) Amino acid sequence alignment between the wild-type and mutant proteins was performed using ESPript 3.0 (https://espript.ibcp.fr/ESPript/cgi-bin/ESPript.cgi)


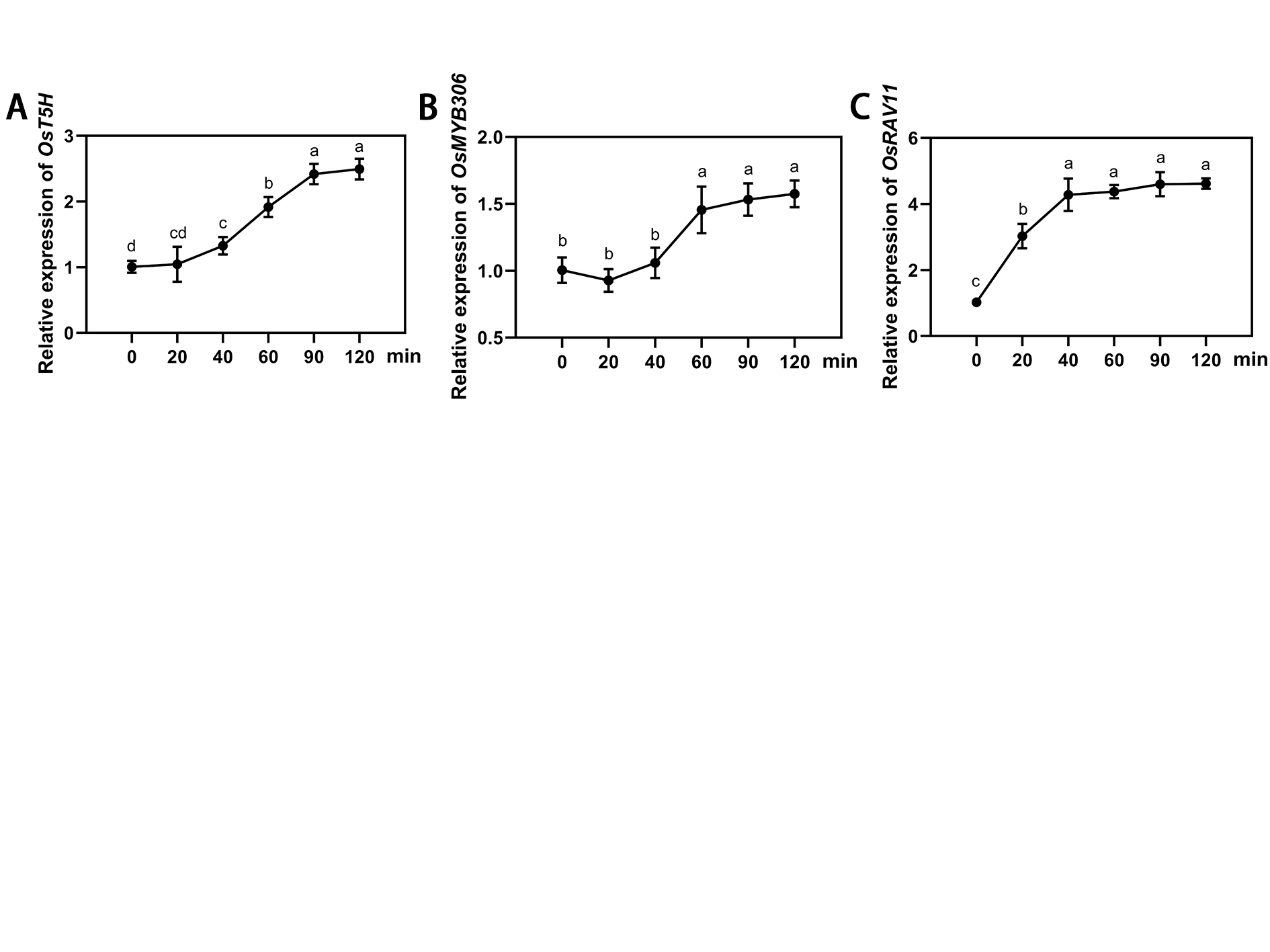
 **Supplemental Figure 9. Temporal dynamics of *OsT5H*, *OsMYB306* and *OsRAV11* in response to mechanical damage.**

Rice plants at the flowering stage were subjected to mechanical damage using a hole puncher. Samples were collected at the indicated time points (20, 40, 60, 90 and 120 min) after treatment, with 0 min serving as the untreated control. The relative transcript levels of *OsT5H* (A), *OsMYB306* (B) and *OsRAV11* (C) were quantified by RT–qPCR. Expression levels are presented relative to the 0 min time point. Data are shown as mean ± SD from three independent biological replicates. Different letters indicate significant difference at P < 0.05 level (Student’s *t*-test).


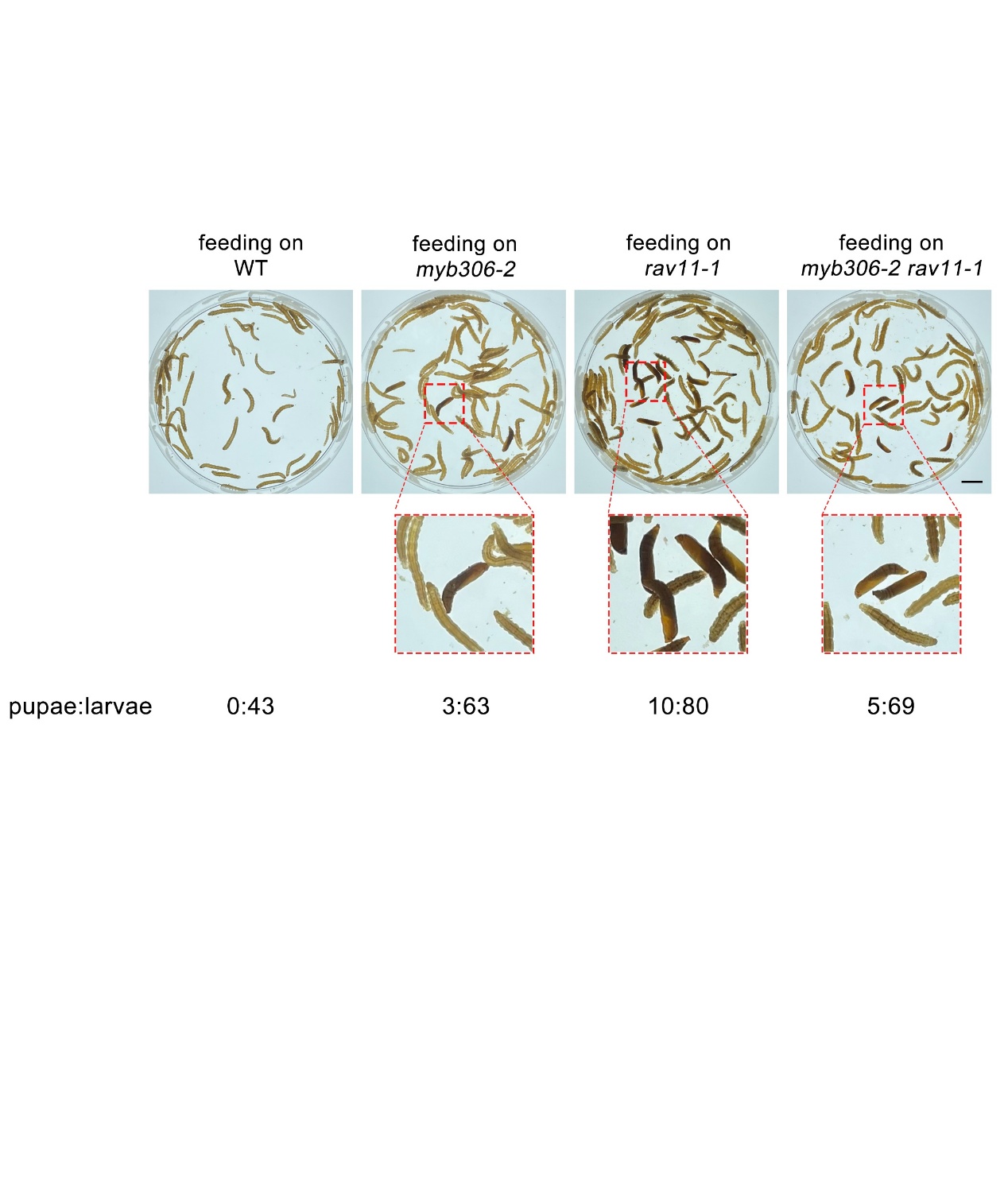
**Supplemental Figure 10. Performance of SSB larvae fed on different materials.**

Performance of 100 SSB larvae fed WT, *myb306-2*, *rav11-1* or *myb306-2 rav11-1* lines 20 days post-infestation. Bottom, the ratio of pupae to larvae. Scale bars, 1 cm.


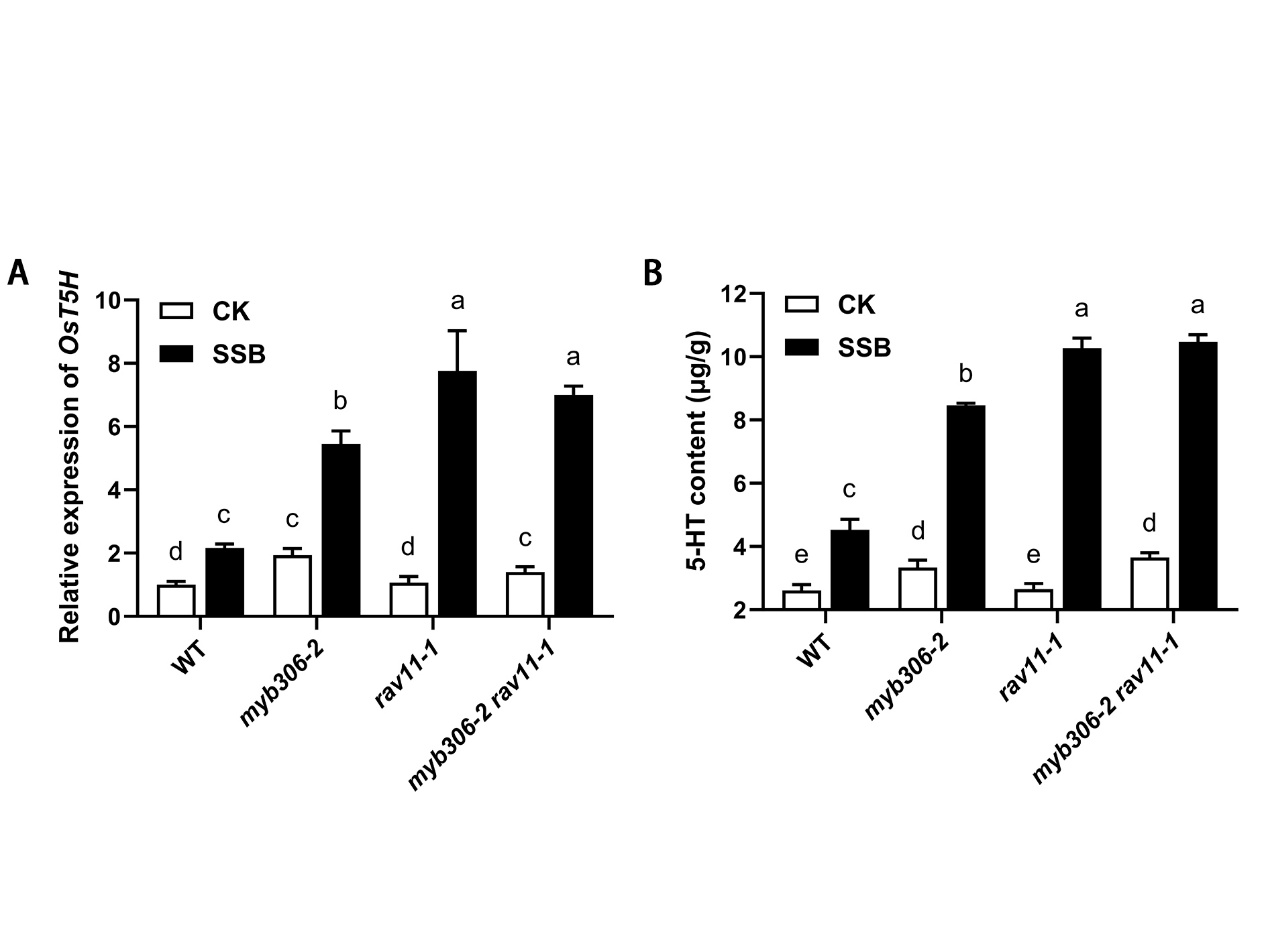
**Supplemental Figure 11. Relative expression of *OsT5H* and 5-HT content in the WT and mutant plants after SSB feeding.**

(A) RT-qPCR analysis of *OsT5H* in 2-month‐old WT, *myb306-2*, *rav11-1* and *myb306-2* *rav11-1* plants with or without 24 h SSB feeding. Data are shown as mean ± SD of three biological replicates. Different letters indicate significant difference at P < 0.05 level (Student’s *t*-test).

(B) 5-HT levels in rice plants as treated in (A). The quantification was performed using HPLC. Data are shown as mean ± SD of three biological replicates. Different letters indicate significant difference at P < 0.05 level (Student’s *t*-test).


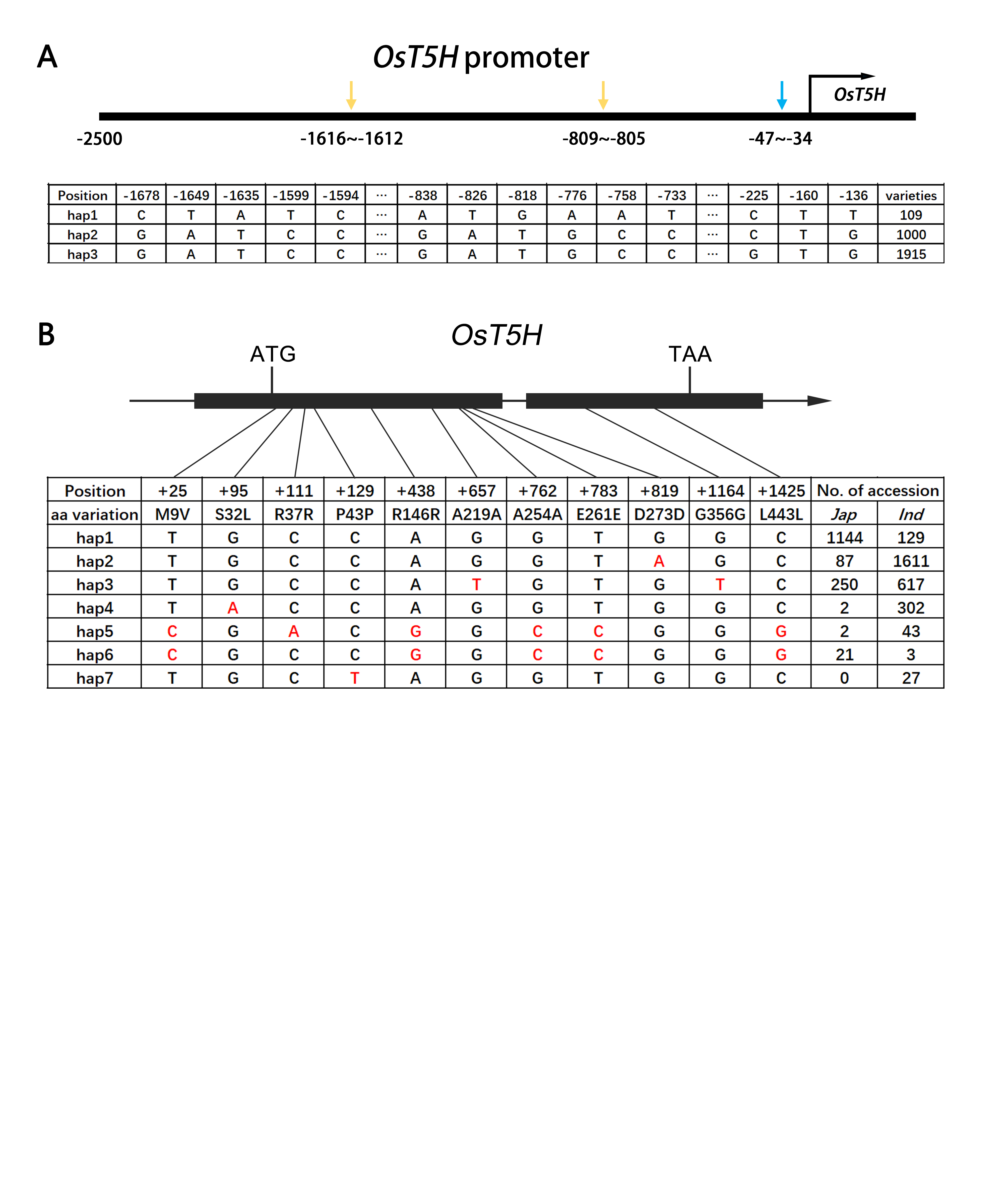
 **Supplemental Figure 12. Natural variation and haplotype analysis of *OsT5H* in the 3k rice collection.**

(A) Sequence variation in the 2500 bp promoter region. The experimentally confirmed binding sites of *OsMYB306* and *OsRAV11* are indicated by blue and yellow arrows, respectively.

(B) Haplotype analysis of the coding region. Seven major haplotypes (hap1-hap7) were identified based on 11 SNPs. The number of accessions carrying each haplotype in *japonica* and *indica* subspecies is indicated on the right.


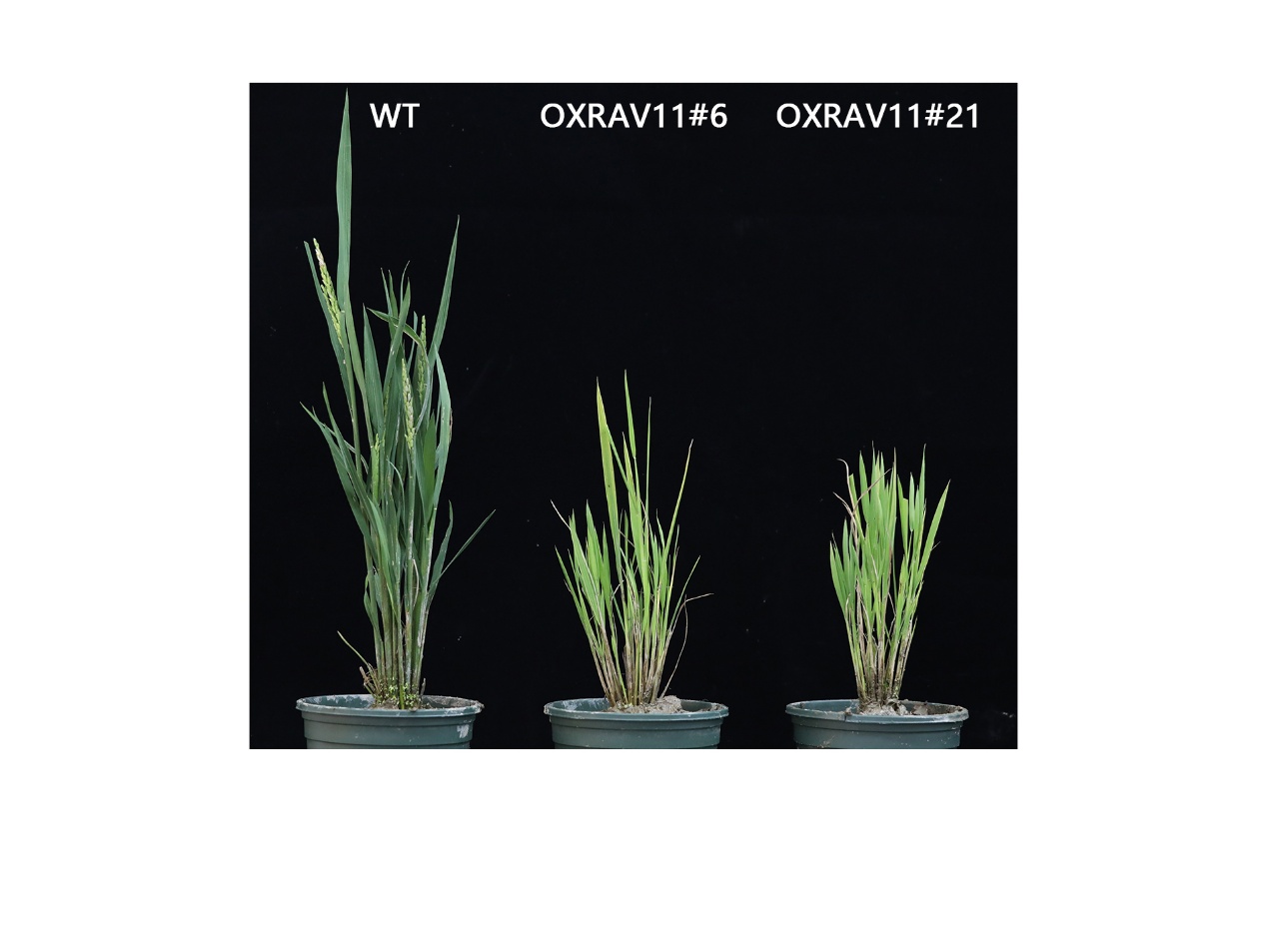


**Supplemental Figure 13. Growth phenotypes of WT and *OsRAV11* overexpression lines.**

Representative plants of WT and two independent *OsRAV11* overexpression lines (OXRAV11#6 and OXRAV11#21) at the heading stage grown under normal conditions.
